# Insights into Red Sea Brine Pool Specialized Metabolism Gene Clusters Encoding Potential Metabolites for Biotechnological Applications and Extremophile Survival

**DOI:** 10.3390/md17050273

**Published:** 2019-05-08

**Authors:** Laila Ziko, Mustafa Adel, Mohamed N. Malash, Rania Siam

**Affiliations:** 1 Graduate Program of Biotechnology, School of Sciences and Engineering, American University in Cairo, New Cairo, Cairo 11835, Egypt; laila.adel@aucegypt.edu (L.Z.); mustafa.adel@aucegypt.edu (M.A.); 2 Biology Department, School of Sciences and Engineering, American University in Cairo, New Cairo, Cairo 11835, Egypt; mnmalash@aucegypt.edu; 3 Microbiology and Immunology Department, Faculty of Pharmacy, Ahram Canadian University, Giza 12581, Egypt

**Keywords:** specialized metabolism gene clusters, Red Sea brine pools, extremophiles

## Abstract

The recent rise in antibiotic and chemotherapeutic resistance necessitates the search for novel drugs. Potential therapeutics can be produced by specialized metabolism gene clusters (SMGCs). We mined for SMGCs in metagenomic samples from Atlantis II Deep, Discovery Deep and Kebrit Deep Red Sea brine pools. Shotgun sequence assembly and secondary metabolite analysis shell (antiSMASH) screening unraveled 2751 Red Sea brine SMGCs, pertaining to 28 classes. Predicted categorization of the SMGC products included those (1) commonly abundant in microbes (saccharides, fatty acids, aryl polyenes, acyl-homoserine lactones), (2) with antibacterial and/or anticancer effects (terpenes, ribosomal peptides, non-ribosomal peptides, polyketides, phosphonates) and (3) with miscellaneous roles conferring adaptation to the environment/special structure/unknown function (polyunsaturated fatty acids, ectoine, ladderane, others). Saccharide (80.49%) and putative (7.46%) SMGCs were the most abundant. Selected Red Sea brine pool sites had distinct SMGC profiles, e.g., for bacteriocins and ectoine. Top promising candidates, SMs with pharmaceutical applications, were addressed. Prolific SM-producing phyla (Proteobacteria, Actinobacteria, Cyanobacteria), were ubiquitously detected. Sites harboring the largest numbers of bacterial and archaeal phyla, had the most SMGCs. Our results suggest that the Red Sea brine niche constitutes a rich biological mine, with the predicted SMs aiding extremophile survival and adaptation.

## 1. Introduction

In the era of antibiotic resistance and a concern for a post-antibiotic era there is a pressing need to combat resistance and discover novel antibiotics [1,2,3]. In the USA alone, around 2 million people a year acquire a bacterial infection that is resistant to all available antibiotics [4]. Additionally, anticancer chemotherapeutic resistance is another recent biomedical challenge, which arises either intrinsically or extrinsically, following therapy [5]. Therefore, it is a necessity to search for new chemotherapeutics [3,4,5].

Nature is considered a mine to explore for small molecules, which may be used as new therapeutic drug leads. Until 2014, a large portion of the rising number of drugs was attributed to natural products [6]. Many organisms and microbes produce specialized metabolites that have a plethora of functions [7]. These microbes’ unique metabolic activities result in the production of specialized metabolites that facilitate their survival in highly competitive environments [7]. Specialized metabolites are mostly coded in the host genomes as clusters of genes, the specialized metabolism gene clusters (SMGCs), which occupy large chunks within prokaryotic genomes [8]. Examples of SMGCs are those encoding for polyketide synthases and non-ribosomal peptide synthases [9]. Compounds with novel chemistry have been identified by prior detection of these biosynthetic gene clusters, such as salinilactam antibiotic (Figure S1A) [10,11]. Some SMGCs are responsible for producing bioactive compounds and have guided the discovery of novel antibiotics [12]. For example, the search for SMGCs in the human microbiome led to the discovery of a new antibiotic named lactocillin (Figure S1B), which is a small molecule produced by a prevalent strain in the human vagina [12]. Several methods were previously developed to detect specialized metabolites and SMGCs, from individual microbes and from communities of microbes [8]. To mine for SMGCs in microbes inhabiting different environments, metagenomic approaches are employed [8]. One approach utilizes environmental DNA isolation, followed by shotgun sequencing and then computational search for SMGCs by bioinformatic tools such as the antibiotics and secondary metabolite analysis shell (antiSMASH) pipeline [9,13,14].

An interesting niche to discover new bioactive compounds is the marine environment, which hosts ample and highly diverse microbes [15]. Previous studies showed that marine organisms produce a large collection of compounds with diverse activities including anticancer and antibacterial agents, such as streptochlorin and abyssomicin C (Figure S1C,D) [16]. Deep-sea microbes are predicted to have a huge biosynthetic versatility as they are able to produce more specialized metabolites of novel chemistry than microbes inhabiting surface seawater [17]. More than 300 new natural products have been isolated from organisms inhabiting the deep sea [17]. Some microorganisms living in extreme deep-sea environments are considered extremophiles. Microbes thriving in more than one extreme condition, i.e. polyextremophiles may thrive under relatively high pressure, salinity, extreme temperatures, scarce nutrients and different pH conditions [18]. The Red Sea brine pools, including Atlantis II Deep (ATII), Discovery Deep (DD) and Kebrit Deep (KD), are known for polyextremophilic conditions [19,20,21,22]. The largest of the 25 Red Sea brine pools is ATII, which is characteristic by hypersalinity (252 psu), high temperature (~67.1 °C) and high metal content [20,23,24,25]. The differential salinity, temperature and pH of the brine water allows the vertical stratification into different layers; an upper convective Layer (ATII UCL), a lower convective Layer (ATII LCL) and brine-seawater interface (ATII INF) [21,26]. DD brine pool is geochemically similar to ATII, as they are in close proximity and have subsurface connections [21,23,24,26]. However, the conditions in the DD brine pool are less harsh than in ATII brine pool [20]. Although KD is not a hot brine, it is characterized by high H_2_S concentration (maximum of 14 mg sulfur/L) [20,23,24,27]. Beneath the brine pools are sediments that are rich in heavy metals [23,28,29]. 

The metabolomes of extremophiles are currently being explored for the discovery of new drugs, and lead to the discovery of new compounds such as salinosporamide K (Figure S1E) [18,30]. Furthermore, Red Sea *Bacillus paralicheniformis* strains were found to harbor more putative biosynthetic gene clusters, and code for potentially bioactive metabolites, such as non-ribosomal peptides [31]. Isolates from Red Sea coastal sediments were reported to have potential antimicrobial activity [32]. Several extremozymes have been isolated and characterized from the ATII Red Sea brine pool as well, such as a thermophilic esterase, a nitrilase and antibiotic-resistant enzymes [33,34,35]. 

In this study we investigated specialized metabolism gene clusters in Red Sea brine water and sediment samples through metagenomic mining. Metagenomes from different sites in the Atlantis II, Discovery Deep and Kebrit Deep brine pools [28,36] were comprehensively studied to determine quantitatively and qualitatively SMGCs in the selected Red Sea brine prokaryotic metagenomes. Red Sea brine SMGCs’ homology to known biosynthetic gene clusters, predicted functions and structures of the encoded potential specialized metabolites are addressed, and their potential contribution in shaping the microbial communities in such harsh environments are discussed.

## 2. Results

### 2.1. Abundance and Diversity of Specialized Metabolism Gene Clusters (SMGCs) in Red Sea Brine Pools

**Figure 1:**
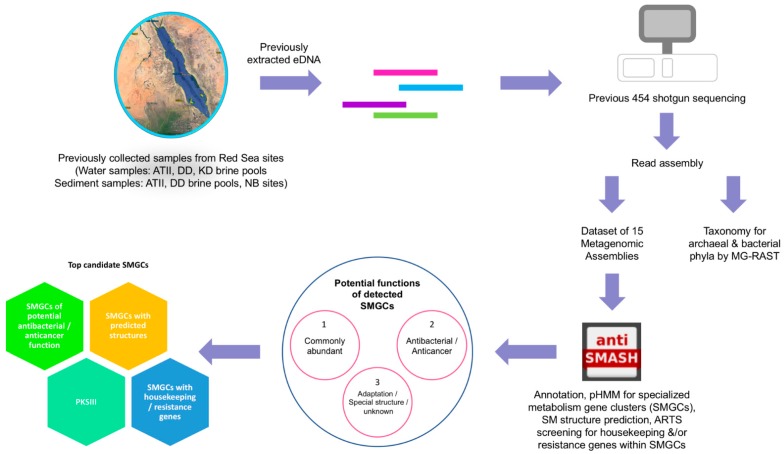
Study workflow for the analysis of specialized metabolism gene clusters in Red Sea brine pools. Water and sediment samples were earlier collected from Red Sea Atlantis II Deep (ATII), Discovery Deep (DD), Kebrit Deep (KD) brine pools and brine-influenced (NBI, NBII) sites. Metagenomic prokaryotic DNA was then extracted from each site and 454 shotgun sequencing was performed followed by read assembly. Taxonomic classification for archaeal and bacterial phyla was performed by protein-based phylogeny using the metagenomics rapid annotation using subsystems technology (MG-RAST) tool [37]. The metabolite analysis shell (AntiSMASH) tool was then used for annotation, for identifying specialized metabolism gene clusters (SMGCs) by translated amino acid sequence comparison with signature biosynthetic genes profile hidden Marcov Model (pHMMs), and for structure prediction of the specialized metabolites [9]. The Antibiotic Resistance Target Seeker (ARTS) tool was used to detect housekeeping and/or resistance genes within the SMGCs [38]. The predicted specialized metabolites were grouped by their potential functions into three major groups. Lastly, top candidate SMGCs were identified in the Red Sea brine dataset.

The study workflow is depicted in Figure 1, and the detailed sample locations are available in Table S1. DNA from Red Sea brine water and sediment samples were previously shotgun sequenced [19,20,22]. In this study we assembled 12,968,227 reads, generating a total of 349,631 contigs (Table 1). The contigs (assembled metagenomes) were used to investigate the SMGCs of the Red Sea brine pools. The assembly metrics are denoted in Table 1. In order to eliminate the effects of assembly size bias in downstream analyses, the number of assembled reads for each site was used to normalize the number of detected SMGCs (Table 1).

ATII LCL had the largest, and ATII 1500 had the smallest number of assembled reads, in the water samples (3,901,967 and 316,101 assembled reads, respectively) (Table 1). Pertaining to the sediment (SDM) samples, DD SDM and ATII SDM cross assemblies comprised the most assembled reads (597,552 and 478,453 assembled reads respectively) (Table 1). NB SDM had the least number of assembled reads amongst all the sites in the dataset (92,530 assembled reads) (Table 1). 

**Figure 2:**
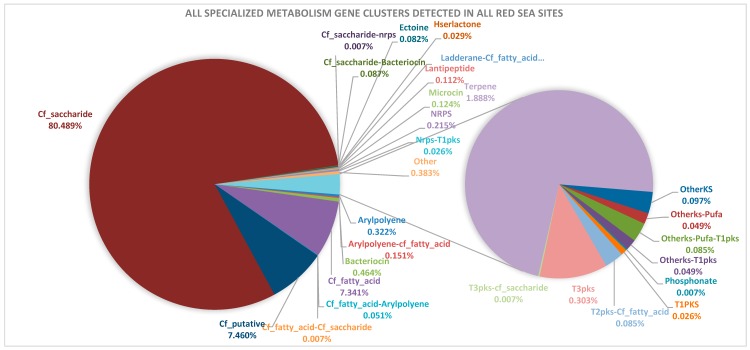
Overview of the specialized metabolism gene clusters encoded by the Red Sea brine pool metagenomes. The detected gene clusters are named as denoted by antiSMASH [9]. Normalized SMGC values were used.

A total of 2751 SMGCs (absolute value) were detected in all the sites by antiSMASH tool (Table 2 and Table S2). The normalized SMGC values are recorded in Table S3. The deepest water samples in the Atlantis II Deep water column samples (ATII 1500 and ATII 700) and the deepest Kebrit Deep brine layer (KD brine–seawater Lower Interface, KD LINF) had the highest number of detected SMGCs; 531.48, 485.27 and 360.56 respectively. The average number of detected SMGCs was 247.03 per site (Table 2). Based on the abundance of SMGCs detected in all sites, three SMGCs cohorts were identified. Cohort 1—representing >7% of total SMGCS: cf_saccharide (80.49%), cf_putative (7.46%) and cf_fatty_acid (7.34%), 2—representing 0.29—7% of total SMGCS: terpenes (1.89%), bacteriocins (0.46%), tther (0.38%), arylpolyene (0.32%), and T3PKS (0.30%) and 3—representing 0.21—0.01% of total SMGCS: NRPS, aryl-polyene-cf_fatty_acid, microcin, lantipeptide, OtherKS, Cf_saccharide-bacteriocin, otherks-Pufa-T1pks, T2pks-Cf_fatty_acid, ectoine, ladderane-Cf_fatty_acid, Cf_fatty_acid-arylpolyene, otherks-Pufa, otherks-T1pks, hserlactone, Nrps-T1pks, T1PKS, Cf_fatty_acid-Cf_saccharide, Cf_saccharide-nrps, phosphonate and T3pks-cf_saccharide (Figure 2). 

The Cf_saccharide gene cluster was the only SMGC detected in all the Red Sea sites. Selected SMGCs were unique to specific sample sites, namely, Cf_fatty_acid-Arylpolyene—was only detected in ATII 200, Otherks-Pufa and Otherks-T1pks were only detected in ATII 700, Otherks-Pufa-T1pks and T2pks-Cf_fatty_acid were only detected in ATII 1500, Cf_fatty_acid-Cf_saccharide and Cf_saccharide-nrps and Phosphonate and T3pks-cf_saccharide were only detected in ATII LCL, Cf_saccharide-Bacteriocin and Hserlactone were only detected in KD LINF and Ladderane-Cf_fatty_acid and Nrps-T1pks and T1PKS were only detected in KD brine–seawater Upper Interface (KD UINF). Additionally, among the non-Red-Sea dataset, Pufa (polyunsaturated fatty acid cluster) was unique to Guaymas Basin deep-sea hydrothermal vent plume water sample (GB VNT), thiopeptide cluster was only detected in Kueishantao shallow-sea hydrothermal vent area water sample KSW VNT and acyl_amino_acids (*N*-acyl amino acid cluster) was only detected in Loki’s Castle deep-sea vent black smoker chimney microbial mat sample (LC MM) (Table 2, Tables S2 and S3).

Our analysis resulted in the detection of 28 distinct SMGCs and the diversity of SMGCs in the Red Sea water and sediment samples was recorded (Figure 2, Table S3). The water samples harbored all of the detected 28 SMGC types and only 7 SMGC types were detected in the sediment samples. In order to compare the diversity of the SMGCs detected in our dataset, the absolute values of the different types of SMGCs were used for comparison (Table 2). KD UINF and ATII LCL sites, showed the highest diversity in SMGC types (13 different SMGC types) while DD brine–seawater interface (DD INF) had the lowest diversity (1 SMGC type) (Table 2).

### 2.2. Red Sea Brine Pool SMGCs Code for Diverse Potential Functions

**Figure 3:**
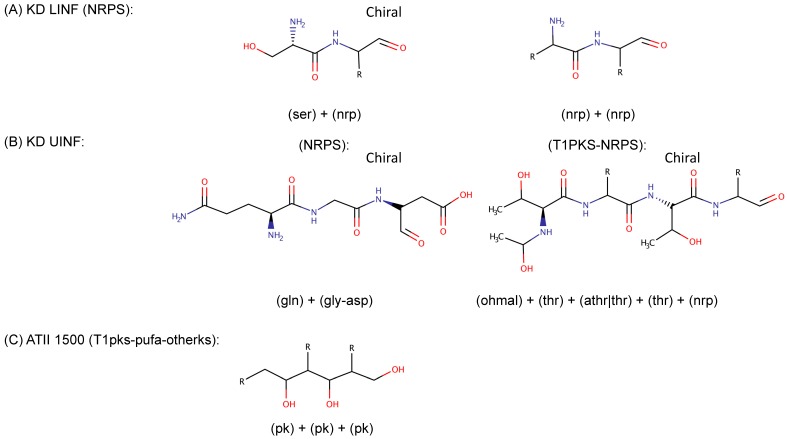

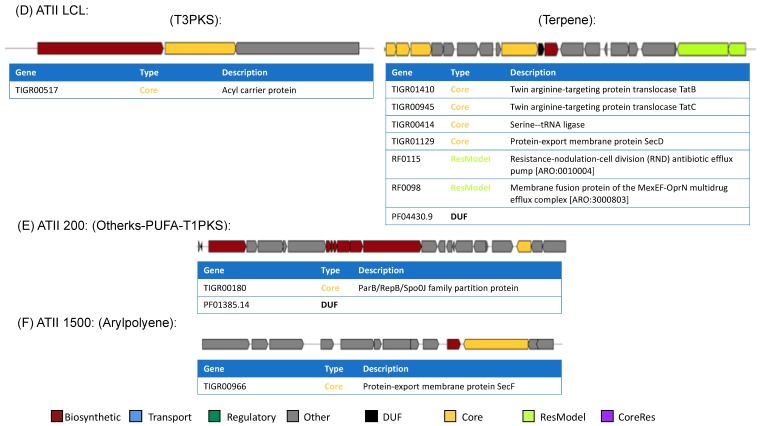
Promising Red Sea brine specialized metabolite candidates. Five core structures for specialized metabolites were predicted using antiSMASH 4.0 [45]: (**A**) KD brine–seawater Lower Interface (KD LINF) site had two non-ribosomal peptides predicted to be synthesized by two non-ribosomal peptide synthetase (NRPS) clusters. (**B**) KD brine–seawater Upper Interface (KD UINF) site had a non-ribosomal peptide predicted to be synthesized by a NRPS cluster and a hybrid polyketide-non-ribosomal peptide predicted to be synthesized by T1PKS-NRPS. (**C**) ATII 1500 site had a polyketide predicted to be produced by a T1pks-pufa-otherks hybrid cluster. Four top promising candidate SMGCs were detected by ARTS [38]: (**D**) ATII brine Lower Convective Layer (ATII LCL) site had two clusters encoding Terpene and T3PKS. (**E**) ATII 200 site had a cluster encoding aryl polyene. (**F**) ATII 1500 site had a cluster encoding Otherks-PUFA-T1PKS (PUFA: polyunsaturated fatty acid). ser: serine, nrp: non-ribosomal peptide, gln: glutamine, gly: glycine, asp: aspartate, thr: threonine, pk: polyketide, ohmal: hydroxy malate.

We classified the predicted Red Sea SMGCs according to the potential function of their products into three main groups: (1) products of predicted functions commonly abundant in microbes, including saccharides, fatty acids, aryl polyenes and acyl-homoserine lactones, (2) subset of products with potential antibacterial and/or anticancer effects, including terpenes, ribosomal peptides, non-ribosomal peptides, polyketides and phosphonates, and (3) miscellaneous products that are predicted to confer adaptation to the environment/special structure/unknown function, including polyunsaturated fatty acids, ectoine, ladderane and others (Table 3). When applicable, the product class representative chemical structures are also depicted in Table 3, e.g., ladderane structure. Additionally, five predicted core structures of the potential products coded by the SMGCs of the Red Sea dataset were computationally predicted (Figure 3). Two core structures were predicted in the KD LINF layer for non-ribosomal peptides, both coded for putative NRPS clusters (Figure 3A). A chiral non-ribosomal peptide structure was predicted in KD UINF layer that was encoded by a putative NRPS cluster, as well as a hybrid polyketide-non-ribosomal peptide chiral structure encoded by a T1PKS-NRPS cluster (Figure 3B). The fifth structure was a polyketide predicted in ATII 1500 layer and was encoded by the putative hybrid cluster T1pks-pufa-otherks (Figure 3C). To prioritize specific SMGCs for further experimental work among all the detected SMGCs, Antibiotic Resistance Target Seeker (ARTS) analysis was used and revealed that four SMGCs harbored neighboring housekeeping and/or resistance genes (Figure 3D–F).

We identified a subset of SMGCs common in brine pool water samples and distinct from the sediment samples and the overlying water column. Six different SMGCs (Bacteriocin, cf_fatty_acid, cf_putative, cf_saccharide, other and T3PKS) were detected only in the ATII brine samples (ATII INF, ATII UCL, ATII LCL) and four SMGC types (Bacteriocin, cf_fatty_acid, cf_putative and cf_saccharide) were common among the KD samples (KD BR, KD LINF, KD UINF). Additionally, subsets of SMGCs were common in the water column overlying ATII; namely, the three SMGC types (cf_fatty_acid, cf_putative and cf_saccharide). Cf_saccharide was the only SMGC type common in both DD BR and DD INF. Note that four SMGCs (cf_fatty_acid, cf_putative, terpene and cf_saccharide) were detected in all of the sediment samples (ATII SDM, DD SDM and NB SDM) (Table S3).

**Figure 4:**
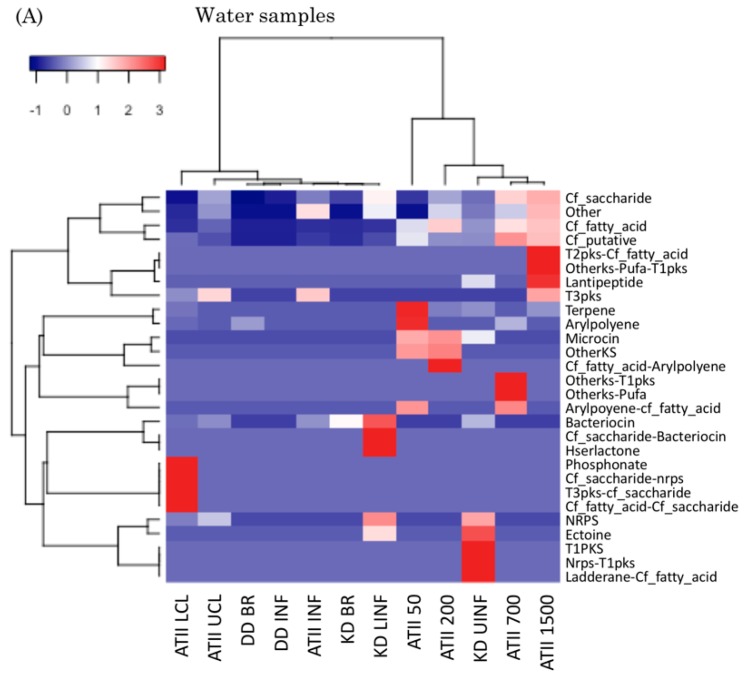

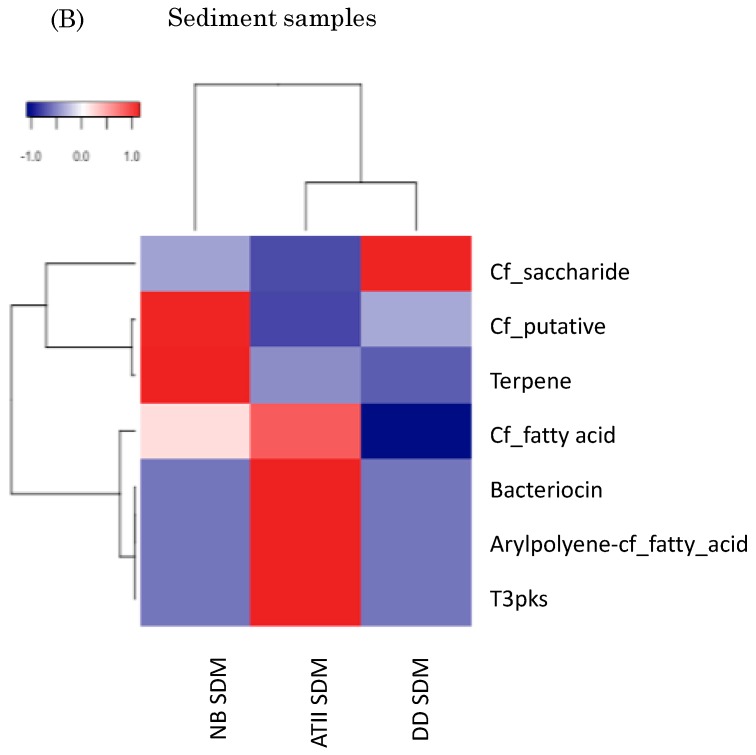
Heat maps representing hierarchical classification of the SMGCs detected in Red Sea brine pool metagenomes. (**A**) Heat map for the Red Sea water samples SMGCs. (**B**) Heat map for the Red Sea sediment samples SMGCs. Hierarchical clustering was based on the relative abundance of normalized numbers of SMGCs detected at each site.

When hierarchical clustering was computed for the water samples only (Figure 4A), i.e. excluding the sediment samples, most brine SMGCs reads grouped together (ATII LCL, ATII UCL, DD BR, DD INF, ATII INF, KD BR and KD LINF), except for KD UINF. Yet, ATII 50-1500 m and KD UINF water samples clustered together (Figure 4A). Hierarchical classification of the sediment samples revealed that brine pool SMGCs clustered together (ATII SDM and DD SDM) (Figure 4B). When all the samples in the dataset were used as an input for hierarchical classification (i.e. Red Sea and non-Red Sea metagenomes), the non-Red Sea SMGCs clustered together as an outgroup to Red Sea samples (Figure S2). 

### 2.3. Red Sea Brine Prokaryotic Diversity in Relation to Specialized Metabolism Genes

**Figure 5:**
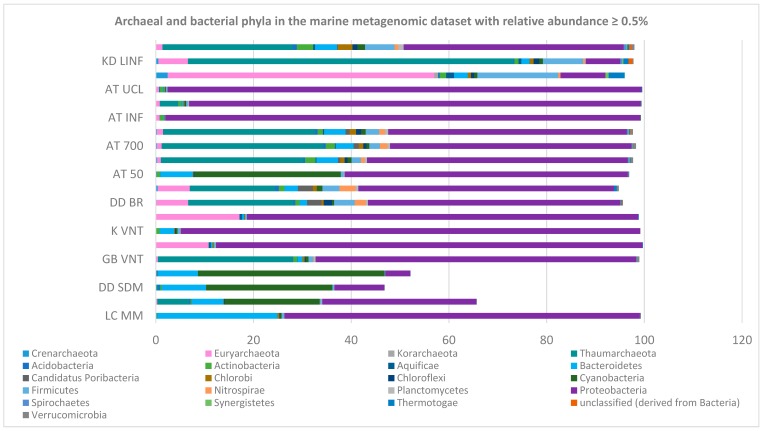
Archaeal and bacterial phyla detected in the marine metagenomic dataset. The relative abundancies (represented as % of total detected phyla) detected by MG-RAST are presented. The phylum is represented if its relative abundance is ≥ 0.5% in at least one of the assemblies.

To correlate the microbial communities and how their taxonomic profiles contribute to specialized metabolites production, archaeal and bacterial phyla were investigated and were related to the SMGCs found in each site (Figure 5, Tables S4–S6). The abundance of major archaeal and bacterial phyla in the dataset was previously reported, albeit for the sequence reads not for the assembled contigs [22,36]. However, in this study we focused on bacterial phyla with the potentiality to produce bioactive products; as certain bacterial phyla were reported to produce bioactive compounds such as antibacterial and anticancer agents. 

A total of 5 archaeal phyla and 28 bacterial phyla were detected in all of the Red Sea assemblies analyzed (Figure 5, Table 2, Tables S4 and S5). ATII 1500 and KD LINF showed the most diverse archaeal and bacterial phyla sites and each harbored all of the detected archaeal and bacterial phyla (33 distinct phyla), while NB SDM showed the least number of bacterial phyla (9 distinct phyla). The following phyla were ubiquitously detected in all the sites included in the dataset: Proteobacteria, Cyanobacteria, Acidobacteria, Actinobacteria, Bacteroidetes, Chlorobi and Firmicutes. Euryarchaeota and Thaumarchaeota were present in all the water samples (Figure 5, Table S4). Sediment samples harbored viruses (33.81%–52.72%) and Cyanobacteria (19.46%–38.18%) in addition to Proteobacteria (5.12%–31.51%), as major taxa. The percentage of the viruses was lower in all the water samples (0.23%–3.96%). DD samples harbored mainly Thaumarchaeota (17.44%–21.50%) and Proteobacteria (51.63%–52.33%) as major taxa. ATII 50 harbored mainly Cyanobacteria (29.97%), while ATII 200-1500 harbored Thaumarchaeota (29.12%–33.08%) and ATII 50-1500 harbored Proteobacteria (48.86%–57.97%) as major taxa. ATII brine layers mainly harbored Proteobacteria (92.44%–97.16%) as the major taxa. KD BR mainly harbored Euryarchaeota (54.59%) while KD UINF-LINF harbored Thaumarchaeota (26.68%–66.62%), and all KD layers harbored Firmicutes (6.06%–16.57%) and Proteobacteria (7.07%–45.04%) as major taxa. The most abundant archaeal and bacterial genera were recorded for each site (Table S6). Additionally, the prokaryotic phyla unique to Red Sea samples were recorded, prokaryotic phyla common to Red Sea and other hydrothermal vent metagenomes as well as the prokaryotic phyla unique to the other hydrothermal vent metagenomes (Table S10).

### 2.4. Rare Leucine Codons within Red Sea Brine SMGCs and Low Similarity to Known Clusters with Characterized Products

The counts of TTA codons were denoted for all the Red Sea SMGCs, excluding saccharides and fatty acids (Table S7). All the Red Sea sites harbored TTA rare leucine codons except DD INF layer, with a total of 7684 (absolute value), and an average of 512. The TTA codon counts were particularly high (>1000) in ATII 50, KD UINF and ATII 700 water layers. 

To detect homologous gene clusters to the SMGC hits found in the Red Sea brine pool dataset we used 1-ClusterBlast, 2- KnownClusterBlast and 3- SubClusterBlast algorithms which are embedded in antiSMASH pipeline, for all the SMGCs except for cf_saccharides and cf_fatty_acids, (Table S8) [45]. Those algorithms detect homologous gene clusters and thus identify taxonomic and functional characteristics for the SMGCs [45]. Out of the 301 SMGCs, 204 were found to be of significant homology to gene clusters in the database, 30 SMGCs had identified homologous known gene clusters while only 4 SMGCs showed significant hits with homologous subclusters (Table S8). 77 of the 301 SMGCs had no significant homologous gene clusters, according to the ClusterBlast module embedded in antiSMASH (Table S8). The percentage of genes with similarity to the Red Sea SMGCs ranged between 2%–43% for the homologous gene clusters, 2–77% for the homologous known gene clusters and 20–66% for the homologous subclusters.

## 3. Discussion

### 3.1. Saccharide and Putative SMGCs Are the Most Abundant Groups in the Red Sea Brine Dataset

Metagenomic read sequences were previously reported for ATII water column samples [19], brine ATII, DD and KD water samples [20], as well as for the sediment samples [36]; 16S rRNA and taxonomic analysis of the different sediment sections were previously discussed [28]. In this study we utilized the assembled metagenomes for all the previously mentioned samples, in order to detect and thoroughly analyze the specialized metabolism gene clusters encoded in the Red Sea brine pool metagenomic dataset.

A total of 2751 SMGCs belonging to 28 different SMGC types, were detected in all 15 assembled metagenomes (Figure 1 and Figure 2). The average number of detected clusters/Mb in the Red Sea dataset was 0.38 SMGC/Mb, ranging from 0.13 SMGC/Mb (NB SDM) to 0.67 SMGC/Mb (KD LINF) (Table 1 and Table 2). The average number of detected SMGC/Mb in more than 1000 studied bacterial genomes was reported to be 2.4 [46], ~6 times higher than that of the Red Sea brine metagenomic dataset. The number of SMGCs detected by antiSMASH is linear to the bacterial genome size [46], however, metagenomic data analysis is inherently more challenging than genomic data, owing to the short reads followed by assembly [61]. This could explain the relatively limited number of detected SMGCs. Recent studies detected SMGCs in genomes of new soil-residing *Pseudovibrio* bacterial strains using antiSMASH [62,63].

The most abundant SMGC class detected in the Red Sea samples coded for saccharides (80.49% of total SMGCs) (Figure 2). This is in concordance with the study of Cimermancic et al., wherein saccharides comprised the largest detected SMGC type (40% of total detected SMGCs) [46]. The second largest group of SMGCs was the cf_putative SMGCs (7.46% of the total SMGCs) (Table S2). Such putative gene clusters are unknown biosynthetic gene clusters, with no specific category assigned to them. It is likely that these novel gene clusters constitute a specific group of ‘SMGC dark matter’ [9], that we aimed to preliminarily categorize.

Selected SMGCs were unique to selected Red Sea sites, e.g. terpene SMGC was common to all SDM samples (Table S3), suggesting that each group of sites possesses a distinct SMGC ‘signature’ profile. Interestingly, ATII LCL has the harshest physicochemical conditions [33] and it harbors the most diverse SMGCs (Table 2), which shows the importance of studying SMGCs in extreme environments. 

### 3.2. Preliminary Evidence of Potential Products with Pharmaceutical Applications

In order to prioritize the search for novel antibacterial and anticancer compounds among the detected orphan SMGCs, we recommend characterizing the following SMGCs in the future: 1, SMGCs coding for terpenes, peptides, polyketides and phosphonates (Table 3, group 2 SMGCs). 2, SMGCs having products with predicted structures (Figure 3A–C), wherein the SMGCs can be expressed, the structures elucidated and their bioactivities characterized [46]. 3, Type III PKSs that are easier to clone than other PKSs, and are capable of producing products with diverse structures [54]. 4, SMGCs likely to encode for novel metabolites, perhaps with antibacterial activity (Figure 3D–F): terpene, type III PKS, aryl polyene and lastly otherks-PUFA-T1PKS. The latter SMGC category should be studied, as core housekeeping genes and/or resistance genes were detected in close proximity to them. Recently, targeted mining genomes was successfully conducted through the detection of resistance and housekeeping genes within SMGCs [38]. Therefore, it is important to further explore those former four SMGCs categories as promising candidate Red Sea brine SMGCs. 

Structural prediction of Red Sea SMGCs products revealed chiral and non-chiral non-ribosomal peptides as well as a polyketide (Figure 3D–F). Only five specialized metabolite core structures were predicted with antiSMASH. Perhaps the Red Sea brine microbiota are evolutionarily distant from the well-characterized genes in the published databases, hindering further product structural and functional prediction. The few predicted Red Sea SMGCs chemical structures (Figure 3D–F) remain to be verified by experimentation e.g. mass spectrometry and comparative metabolomic studies [8]. 

Rare TTA leucine codons are especially enriched in specialized metabolism genes and cell differentiation genes; however, they should be optimized for successful expression [45]. The TTA codon counts were particularly high within ATII 50, KD UINF, and ATII 700 SMGCs (Table S7). This is a possible translational impediment in the case of SMGCs in heterologous hosts, especially for those three sites, and should be accounted for prior to expression.

This metagenome mining study has several limitations because: 1, biosynthetic genes might have been missed as antiSMASH searches for partial or complete gene clusters rather than individual genes, 2, small contigs were excluded as the contig length cut-off was 1000 bp, and 3, antiSMASH detects SMGCs using a rule-based approach based only on the known pathways, so it is likely that we obtained less hits, e.g., SMGCs utilizing un-characterized pathways [64].

Functional screening of the ATII LCL fosmid library lead to the detection of orphan biosynthetic gene clusters that conferred antibacterial and anticancer effects [65]. Hence both functional [65] and computational detection of SMGCs in Red Sea brine pools corroborate that investigating the Red Sea brine pool niche for SMGCs of pharmaceutical application, is a promising approach.

### 3.3. Red Sea Brine SMGCs form a Unique Cluster

Hierarchical clustering revealed that almost all brine water samples (ATII LCL, ATII UCL, DD BR, DD INF, ATII INF, KD BR and KD LINF) formed a unique SMGC brine cluster (Figure 4A), while KD UINF and ATII 200–1500 water samples clustered together. The physical conditions in KD UINF resembles deep Red Sea water, as the conditions are not as harsh as other brine sites [20,27]. Also, the SMGC percentage composition of KD UINF was closer to ATII 50–1500 m water sites, e.g. cf_saccharide abundance is similar in KD UINF and ATII water samples (77.78% in KD UINF and 58.22%–76.79% in ATII water samples), as opposed to 92.51% and 95.30% in the other KD samples. The detailed percent composition of each SMGC per Red Sea brine site is included (Table S9). Brine sediments had similar SMGC profiles, perhaps due to similar environmental conditions as opposed to the NB site, the latter having the least harsh conditions (Figure 4B) [28]. Upon hierarchical classification of all of the included sites, the Red-Sea metagenomic samples clustered together, contrast with the non-Red Sea SMGC profile, re-emphasizing the possibility of a Red Sea SMGC profile signature (Figure S2).

### 3.4. Environment–Microbe Interaction, Antagonistic Stressors and Extremophile Survival Implicated by Red Sea Brine SMGCs

Certain sites harbored unique SMGCs (Table 2) such as ladderane, that was detected only in KD UINF. Ladderane is exclusively present in the membranes of anaerobic ammonia-oxidizing (anammox) bacteria [66]. Noting that anammox bacteria thrive in the presence of sulfide influx and can perform denitrification [67], and KD is characterized by high H_2_S content [23], thus denitrification may be coupled to sulfide in KD UINF anammox bacteria.

Bacteriocin SMGCs were detected in all ATII and KD brine water samples (Table S3), which are putative antagonistic stressors. An earlier study detected numerous halocins–bacteriocins and archaeocins produced by halophiles and were hypothesized to play a role in ensuring microbial diversity in hypersaline environments [68]. Microbes inhabiting ATII and KD niches seem to be utilizing similar mechanisms for survival. KD samples also hosted ectoine synthase-coding genes in the brine–seawater interface layers (Table S3). Ectoine is a stress solute that enables halophiles to withstand high salt content [59], and thus “salt-out” mechanism is likely to account for KD microbes survival in such high salinity [69].

Terpene SMGCs were detected in all SDM samples (Table S3). A previous study suggested that diterpenoids produced by the hyperthermophilic *Chloroflexus aurantiacus* may function in modulating membrane fluidity [70]. It is likely that terpenes provide the correct cell membrane fluidity for extremophiles [70]. Further experimentation is needed to shed light on the particular structure and function of the Red Sea terpenes. Terpenes with novel chemistries were isolated from a fungus inhabiting deep-ocean sediments, and conferred anticancer activity [71]. Terpene SMGCs were most enriched in the ATII 50 site, pointing to the possibility that shallow seawater favors terpene production. ATII brine water layers all harbored PKSIII SMGCs (Table S3). PKSIII enzymes are capable of producing different chemical structures, some of which have important bioactivities [54]. Maybe different stress factors within the same brine, ATII, have interfered in the overlaying water and underlying sediment microbiomes to produce the terpene SMGC signature, while causing the brine water layer microbiomes to have the PKSIII SMGC signature.

### 3.5. Prolific Specialized-Metabolite-Producing Phyla Detection and Red Sea Brine Pool SMGC Dark Matter Analysis

As expected, all Red Sea sites harbored Proteobacteria, Actinobacteria and Cyanobacteria (Table S5). Yet these phyla are known to produce a huge repertoire of specialized metabolites [46,72,73,74]. Certain bacterial taxa produce above average SMGCs/Mb, e.g., *Myxococcus*, *Streptomyces* and *Gloeobacter* –which belong to the aforementioned phyla, respectively [46]. Our analysis of the Red Sea brine dataset revealed that not all the phyla were shared in neighboring sites, even when their SMGCs clustered together, e.g., KD BR and KD LINF. Additionally, not all neighboring sites clustered together based on the SMGCs even if they harbored similar phyla, e.g., KD UINF and KD LINF (Figure 4 and Figure 5). These observations hint at a debatable relationship between SMGCs and phylogeny, similar to that reported for genomic datasets [46].

Members of the most abundant bacterial and archaeal genera (Table S6) were reported to be living in similar niches or having biotechnological applications, e.g., *Nitrosopumilus maritimus* survives in minimal ammonia available in its marine environment [75], and *Prochlorococcus MIT9313* produces lanthipeptides [76]. Only 10% of the SMGCs were homologous to experimentally characterized gene clusters (Table S8), indicating a huge opportunity to further investigate the Red Sea brine pool SMGC dark matter. Several homologous gene clusters pertained to aquatic microbes (e.g., *Mizugakiibacter sediminis*) [77], while others pertained to halophilic microbes (e.g., *Verrucomicrobia bacterium*) [78]. SMGC similarity may contribute to microbial evolution in similar ecological niches [79], perhaps leading to a future halophilic SMGC signature profile.

When we compared the phyla in the Red Sea samples to the other included marine hydrothermal vents (GB VNT, KSW VNT, K VNT, JDF VNT, LC MM), we identified 145 unique prokaryotic phyla in the Red Sea samples (Table S10). The unique phyla to the Red-Sea, the unique phyla to the other marine hydrothermal vent metagenomes and the common phyla in all sites used are presented (Table S10).

## 4. Materials and Methods

### 4.1. Sampling, DNA Extraction and Sequencing

The overall study workflow that we employed is depicted in Figure 1. The Red Sea samples analyzed included ATII brine water samples (ATII INF: ATII brine–seawater Interface, ATII UCL: ATII brine Upper Convective Layer, ATII LCL: ATII brine Lower Convective Layer), ATII water column samples (ATII 50: ATII 50 m depth, ATII 200: ATII 200 m depth, ATII 700: ATII 700 m depth, ATII 1500: ATII 1500 m depth), DD brine water samples (DD BR: DD brine water, DD INF: DD brine–seawater interface) and KD brine water samples: (KD BR: KD brine water, KD LINF: KD brine–seawater Lower Interface, KD UINF: KD brine–seawater Upper Interface). All the sites are described in [19,20]. Additionally, sediment samples included sediments underlying ATII brine pool (ATII SDM) were included, comprising seven distinct layers, sediments underlying DD brine pool (DD SDM), comprising seven distinct layers and sediments underlying two brine-influenced sites (NB SDM) [28]. In total, 28 different samples were analyzed, including 12 water samples and 16 sediment samples, that were previously described [19,20,28].

The samples were previously collected in April 2010 on *Aegaeo* research vessel that was the second leg of the Red Sea expedition of KAUST/WHOI/HCMR (KAUST: King Abdullah University of Science and Technology, WHOI: Woods Hole Oceanographic Institution, HCMR: Hellenic Center for Marine Research) [19,20,28]. Further information on sample locations are tabulated in Table S1. The water samples were sequentially filtered through 3.0, 0.8 and 0.1 µm filters. DNA was then extracted from the 0.1 µm filter [28]. A Genome Sequencer (GS FLX) pyrosequencer was used for sequencing the DNA samples by Titanium pyrosequencing kit (454 Life Sciences). Quality control for the obtained reads was done by PRINSEQ-lite v0.20.4 [80] and CD-HIT-454 [81].

Five other non-Red-Sea marine metagenomes were additionally selected, pertaining to marine hydrothermal vent samples of varying temperatures. GB VNT: Guaymas Basin deep-sea hydrothermal vent plume water sample (3 °C) [39,40,41]. Water samples from Kueishantao shallow-sea hydrothermal vent area: KSW VNT: vent sample (30 °C), K VNT: water directly above the vent (49 °C) [42]. JDF VNT: Juan de Fuca Ridge hydrothermal vent diffuse flow seawater sample, particularly from the Hulk vent (125 °C) [43]. LC MM: Loki’s Castle deep-sea vent black smoker chimney biofilm sample (306 °C) [44].

### 4.2. Bioinformatics Assembly

The assembly files were generated using Newbler assembler v2.6 [82]. For the Red Sea samples, default parameters were used for overlap layout consensus, except extension over read tips, that was opted to cope with the low coverage in direct DNA 454 shotgun sequencing runs on the metagenomic samples. The reads were limited to one contig in output. For the non-Red Sea samples, default parameters were used.

Distinct assembly files were generated for all the water samples, from particular sites and depths [19,20]. However, sediment (SDM) reads from the same brine pool were cross-assembled, i.e., ATII SDM cross-assembly comprised samples obtained from seven different ATII sediment depths, and DD SDM cross-assembly from seven different DD sediment depths [36]. NB sediments cross-assembly was derived from samples obtained from two brine-influenced sites [28]. Although NB SDM contains sediments from two distinct neighboring sites that are brine-influenced, which are spatially different, they were pooled to provide an outgroup for the brine sediments and serve as non-brine sediment samples. This resulted in the generation of 15 assembly files that were further used for downstream analyses. In addition, five additional assembly files were constructed for the non-Red-Sea samples from other marine hydrothermal vent sites (JDF VNT, KSW VNT, K VNT, LC MM, GB VNT).

### 4.3. Annotation, SMGCs Analyses and Hierarchical Classification

The metagenomic assembly files were run on antiSMASH using contigs of size equal to or larger than 1000 bp. Annotation was performed using the Prodigal gene finding option for metagenomes. The SMGCs were detected by comparison of translated amino acid sequences with pHMM signature for biosynthetic genes using the ClusterFinder algorithm [9]. The full SMGC detection analysis was performed during 08/2015 (i.e. by antiSMASH version 3.0 [9]). All the counts of detected SMGCs were normalized by dividing each value with the number of assembled reads at each site *10^6^ and used for subsequent analyses. To account for the variability in the sequencing depth, normalization was performed. 

In order to detect homologous gene clusters to all Red Sea brine SMGCs—excluding putative clusters for saccharides and fatty acids—the algorithms ClusterBlast, KnownClusterBlast and SubClusterBlast were used. The first algorithm compares the SMGCs with all gene clusters in Genbank database of NCBI (National Center for Biotechnology Information) and its output is useful to identify the closest organism based on SMGCs [45]. The second algorithm compares the SMGCs with the gene clusters that are experimentally characterized in MIBiG database, thus giving an indication on the products likely to be synthesized by the SMGCs [45]. The third algorithm detects operons that are conserved and with a known function and gives more specific information on the product synthesized by the SMGCs [45]. This analysis was performed during 11/2017. Putative clusters coding for the production of saccharides and fatty acids were excluded because the scope of the study was to investigate products of potential ecological functions and/or pharmaceutical applications. The TTA codons were also recorded for each site and the absolute counts and normalized values were denoted for each assembly file. Additionally, predicted core structures of the specialized metabolites were recorded. All the aforementioned analyses were done using antiSMASH version 4.0 [45]. The contigs coding for products other than saccharides and fatty acids were screened for housekeeping and resistance genes within the SMGCs using the ARTS program [38].

Hierarchical classification for all the SMGCs detected in all the assembly files, was performed using R version 3.3.1 (R Development Core Team 2016). Distinct heat maps were generated for water and sediment samples using as an input the normalized SMGC count for each of the sites.

### 4.4. Taxonomic Classification

Taxonomical trees for the archaeal, bacterial and viruses phyla were generated by metagenomics rapid annotation using subsystems technology (MG-RAST) tool using the assembled contigs as the input sequences, by comparing them to the non-redundant protein database M5NR (maximum e-value 1e-5, minimum identity of 70%, minimum alignment length of 50 measured in amino acids for protein and bp for RNA databases) [37,83]. Based on the protein-based phylogeny, the detected phyla were denoted for each site, with focus on the archaeal and bacterial phyla of relative abundance ≥0.5% in at least one of the assemblies.

## 5. Conclusions

Our study highlights the importance of Red Sea brine pool water and sediment microbes and their potential capability in producing specialized metabolites. ATII, DD and KD brine pool sites included in the study are thus worthy of bioprospecting (Figure 2, Table 3). The diverse potential functions of the detected SMGCs’ products in the Red Sea dataset, varying from halophilic adaptations to polyketides and peptides of potential antibacterial activity, renders it an attractive mine for such exploration. Our data provides insights on Red Sea brine SMGCs particularly focusing on antibacterial and anticancer exploration. Promising SMGCs code for products with reported antibacterial and anticancer effects, namely terpenes, peptides, polyketides and phosphonates. Also interesting are SMGCs with predicted structures, and SMGCs harboring housekeeping and/or resistance genes. Cloning such genes clusters would provide information on new ‘cryptic’ gene clusters that might be responsible for synthesis of novel natural products, and improve our understanding of the evolution of extremophiles and their adaptation mechanisms to such extreme environments [84]. Future studies are required to clone and express those SMGCs, in order to elucidate the novel chemical entities that possibly serve as antibacterial and/or anticancer drugs, among other different potential functions. 

## Figures and Tables

**Table 1:** Assembly metrics denoted for each of the Red Sea brine pool and other hydrothermal vent samples.

Description	Detailed Description	Sites	Reference Sampling & Read Sequences/Assembly	Number of Reads	Number of Reads after Trimming	Number of Assembled Reads	Number of Contigs > 500 bp	Average Contig Size (bp)	Largest Contig Size (bp)
Atlantis II water column (Water above brine pool)	Atlantis II 50 m water column	ATII 50	[19]/This study	1,461,910	1,461,904	582,768	36,262	1149	21,887
	Atlantis II 200 m water column	ATII 200		1,260,578	1,260,561	530,441	34,640	1131	25,392
	Atlantis II 700 m water column	ATII 700		1,128,514	1,128,507	554,335	32,860	1285	33,783
	Atlantis II 1500 m water column	ATII 1500		833,739	833,730	316,101	20,374	1331	51,927
Atlantis II brine water	Atantis II brine-seawater interface	ATII INF	[20]/This study	832,138	832,128	743,064	9933	1214	25,150
	Atantis II brine Upper Convective Layer	ATII UCL		886,030	886,019	794,715	11,994	1454	103,389
	Atantis II brine Lower Convective Layer	ATII LCL		4,104,966	4,104,994	3,901,967	19,165	2084	350,936
Discovery Deep brine water	Discovery Deep brine-seawater interface	DD INF	[20]/This study	1,095,181	1,095,157	752,025	14,144	1201	28,080
	Discovery Deep brine water	DD BR		1,111,044	1,111,032	763,387	15,306	1216	22,118
Kebrit Deep brine water	Kebrit Deep Upper brine-seawater interface	KD UINF	[20]/This study	1,562,521	1,562,512	1,020,749	24,517	1495	58,542
	Kebrit Deep Lower brine-seawater interface	KD LINF		1,510,272	1,510,262	926,337	31,983	1241	38,825
	Kebrit Deep brine water	KD BR		1,379,832	1,379,814	913,803	22,280	945	14,864
Other water metagenomes	Guaymas Basin deep-sea hydrothermal vent plume water	GB VNT	[39,40,41]/This study	628,619	628,569	155,841	7654	1082	17,353
	Kueishantao shallow-sea hydrothermal vent (water above vent)	KSW VNT	[42]/This study	261,446	261,399	199,237	411	4685	179,360
	Kueishantao shallow-sea hydrothermal vent (water)	K VNT	[42]/This study	444,655	444,597	338,480	2194	1843	88,498
	Juan de Fuca Ridge hydrothermal vent diffuse flow seawater	JDF VNT	[43]/This study	226,981	226,916	35,357	9366	1135	9366
Sediments	Atlantis II sediments	ATII SDM	[28,36]/This study	1,138,406	1,138,381	478,453	30,352	1194	33,674
	Discovery Deep sediments	DD SDM		1,258,290	1,258,273	597,552	38,529	1233	38,081
	Non-brine sediments	NB SDM		253,568	253,564	92,530	7292	1177	1315
Other metagenome (biofilm)	Loki’s Castle deep-sea vent biofilm (microbial mat)	LC MM	[44]/This study	717,550	717,135	525,719	7897	1546	42,387
Total				22,096,240	22,095,454	14,222,861	377,153	-	-

**Table 2:** SMGCs abundance and the counts of archaeal and bacterial phyla in each site. SMGCs are named using the same acronyms as those used by by antiSMASH tool [45]. Hyphens indicate hybrid clusters. Cf_fatty_acid: fatty acid putative cluster. Cf_saccharide: saccharide putative cluster. Hserlactone: cluster coding for homoserine lactone. NRPS: cluster coding for non-ribosomal peptide synthetase. OtherKS: cluster coding for other types of polyketide synthases. Pufa: cluster coding for poly-unsaturated fatty acids. T1pks: type I polyketide synthase. T2pks: type II polyketide synthase. T3pks: type III polyketide synthase. Acyl_amino_acids: cluster coding for N-acyl amino acid.

Detailed Description	Assembly	Number of SMGCs	Normalized Number of SMGCs *	Types of SMGCs	Number of Phyla	SMGCs Detected Uniquely Once at a Particular Site
Red Sea metagenomic samples:						
Atlantis II 1500 m water column	ATII 1500	168	531.48	9	33	Otherks-Pufa-T1pks, T2pks-Cf_fatty_acid
Atlantis II 700 m water column	ATII 700	269	485.27	8	32	Otherks-Pufa, Otherks-T1pks
Kebrit Deep Lower brine-seawater interface	KD LINF	334	360.56	9	33	Cf_saccharide-Bacteriocin, Hserlactone
Atlantis II 200 m water column	ATII 200	170	320.49	8	30	Cf_fatty_acid-Arylpolyene
Atantis II brine Upper Convective Layer	ATII UCL	210	264.25	7	21	
Atlantis II 50 m water column	ATII 50	146	250.53	8	30	
Kebrit Deep Upper brine-seawater interface	KD UINF	252	246.88	13	32	Ladderane-Cf_fatty_acid, Nrps-T1pks, T1PKS
Atantis II brine-seawater interface	ATII INF	162	218.02	6	19	
Discovery Deep sediments	DD SDM	114	190.78	4	23	
Non-brine sediments	NB SDM	16	172.92	4	9	
Kebrit Deep brine water	KD BR	149	163.05	4	23	
Atlantis II sediments	ATII SDM	70	146.30	7	26	
Atantis II brine Lower Convective Layer	ATII LCL	524	134.29	13	25	Cf_fatty_acid-Cf_saccharide, Cf_saccharide-nrps, Phosphonate, T3pks-cf_saccharide
Discovery Deep brine-seawater interface	DD INF	94	125.00	1	20	
Discovery Deep brine water	DD BR	73	95.63	2	20	
Other metagenomic samples:						
Guaymas Basin deep-sea hydrothermal vent plume water	GB VNT	11	70.58	3	30	Pufa
Kueishantao shallow-sea hydrothermal vent (water above vent)	KSW VNT	11	55.21	4	12	Thiopeptide
Kueishantao shallow-sea hydrothermal vent (water)	K VNT	10	29.54	5	13	
Juan de Fuca Ridge hydrothermal vent diffuse flow seawater	JDF VNT	3	84.85	3	14	
Loki’s Castle deep-sea vent biofilm (microbial mat)	LC MM	26	49.46	5	23	Acyl_amino_acids

* Normalized number of SMGCs are the number of SMGCs detected at each site divided by the number of assembled reads *10^6^.

**Table 3:** The potential functions of the array of specialized metabolites encoded by Red Sea brine SMGCs. SMGCs are named as denoted by antiSMASH tool [45]. Hyphens indicate hybrid clusters. Cf_fatty_acid: fatty acid putative cluster. Cf_putative: unknown type putative cluster. Cf_saccharide: saccharide putative cluster. Hserlactone: cluster coding for homoserine lactone. Lantipeptide: cluster coding for lanthipeptide. NRPS: cluster coding for non-ribosomal peptide synthetase. OtherKS: cluster coding for other types of polyketide synthases. Pufa: cluster coding for poly-unsaturated fatty acids. T1pks: type I polyketide synthase. T2pks: type II polyketide synthase. T3pks: type III polyketide synthase.

General Functional Classification:	Product (Enzyme)		Gene Cluster Names	Representative Basic Structure	Potential Function/Application of Product	Percentage of Total SMGCs
1. Products of predicted functions commonly abundant in microbes	Saccharide		Cf_saccharide Cf_saccharide-Bacteriocin Cf_saccharide-nrps Cf_fatty_acid-Cf_saccharide T3pks-cf_saccharide	-	Microbe-host interactions e.g. lipopolysaccharides. Some saccharides that are diffusible were reported to have antibacterial activities [46,47].	80.61%
	Fatty Acid		Cf_fatty_acid Arylpolyene-cf_fatty_acid Cf_fatty_acid-Arylpolyene Cf_fatty_acid-Cf_saccharide Ladderane-Cf_fatty_acid T2pks-Cf_fatty_acid	-	Structural functions and reported that composition can change as an adaptation to temperature and pressure in the environment [48].	7.69%
	Aryl polyenes		Arylpolyene Arylpolyene-cf_fatty_acid Cf_fatty_acid-Arylpolyene	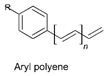	Aryl polyene SMGCs found in abundance in Gram negative bacteria. Previously reported to have a protective role from damage caused by reactive oxygen species [46,49].	0.52%
	Acyl-homoserine lactones		Hserlactone	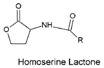	Quorum sensing [50].	0.03%
2. Subset of products with potential antibacterial and/or anticancer effects:	Terpenes		Terpene	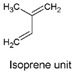	A subset of the terpenes possesses antibacterial effect [51].	1.89%
	Peptides	Ribosomal peptides	Bacteriocin Cf_saccharide-Bacteriocin Microcin Lantipeptide	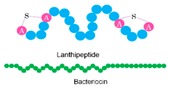	Some have antibacterial activity, and some have selective cancer cytotoxic activity [52].	0.78%
		Non-ribosomal peptides	Cf_saccharide-nrps NRPS Nrps-T1pks	-	Many non-ribosomal peptides have antibacterial (e.g., β-lactams) and anticancer (e.g. bleomycin) effects [53].	0.25%
	Polyketides	(Type I Polyketide synthase)	Nrps-T1pks Otherks-Pufa-T1pks Otherks-T1pks T1PKS	-	A subset are responsible for antibiotic synthesis e.g. the type I polyketide synthase (PKSI) producing erythromycin [54].	0.2%
		(Type II Polyketide synthase)	T2pks-Cf_fatty_acid	-	Some type II polyketide synthase (PKSII) enzymes produce aromatic polyketide antibiotics e.g. oxytetracycline [54].	0.09%
		(Type III Polyketide synthase)	T3pks T3pks-cf_saccharide	-	Type III Polyketide synthase (PKSIII) enzymes are capable of producing an array of compounds including pyrones—a subset of pyrones were previously reported to have antibacterial and anticancer effects [55].	0.31%
	Phosphonates		Phosphonate	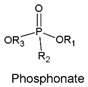	Some natural phosphonates are antibacterials e.g. fosfomycin. Also have structural functions [56].	0.01%
3. Miscellaneous: products are predicted to confer adaptation to the environment/special structure/unknown function:	Others		Cf_putative Other OtherKS Otherks-Pufa Otherks-Pufa-T1pks Otherks-T1pks	-	Some code for biosynthetic gene clusters of types that are still unknown [9].	8.13%
	Polyunsaturated fatty acids		Otherks-Pufa Otherks-Pufa-T1pks	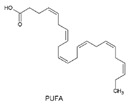	Polyunsaturated fatty acids (PUFAs) are membrane adaptations to piezophiles, thermophiles and psychrophiles to prevent membrane crystallization [57,58].	0.14%
	Ectoine		Ectoine	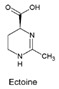	Halophilic adaptation & effective in vitro in preventing protein misfolding characteristic in diseases e.g. Alzheimer’s [59].	0.08%
	Ladderane		Ladderane-Cf_fatty_acid	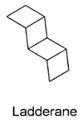	Unique component of anammoxosome membrane in anammox (anaerobic ammonium oxidizing) bacteria and potential biofuel [60].	0.05%

## Data Availability

All the metagenomes included in this study are publicly available on Sequence Read Archive website (SRA: https://www.ncbi.nlm.nih.gov/sra). For each file, the bio-project and sample accession numbers are presented in Table S1.

## Supplementary Materials

The following are available online at https://www.mdpi.com/1660-3397/17/5/273/s1, Figure S1: Chemical structures of some examples of earlier characterized antibiotic and anticancer specialized metabolites: (A) salinilactam, (B) lactocillin, (C) streptochlorin, (D) abyssomicin C and (E) salinosporamide K. Figure S2: Heat map representing hierarchical classification of the SMGCs detected in all the metagenomes in the dataset. Table S1: The sampling locations of each of the sites in the dataset. Table S2: The SMGCs detected at each site (absolute values) with the name of the SMGCs detected, the count and total numbers for each site and for each SMGC are denoted. Table S3: The normalized number of SMGCs detected at each site (normalized: number of SMGCs detected/ number of assembled reads at each site *10^6^), with the name of the SMGCs detected, the count and total numbers for each site and for each SMGC are denoted. In dark blue are the cells with unique SMGCs –only detected once in all the data. Similarly colored cells indicate the SMGCs common to groups of sites (light pink: ATII water column depths, light blue: sediments, yellow: ATII brine layers, bright pink: KD brine layers). Table S4: Archaeal and Bacterial and viral phyla detected for each of the Red Sea brine assembly files. The relative abundance is shown whenever detected to be ≥ 0.5% in at least one of the assemblies. Table S5: All the detected archaeal and bacterial phyla in all the Red Sea brine sites by MG-RAST. The relative abundance is shown for all the phyla and values > 0 are highlighted in red. Table S6: The most abundant archaeal and bacterial genera in all the sites in the dataset. Table S7: Table showing the rare leucine TTA codon absolute and normalized count in each site, for all the Red Sea brine SMGCs excluding the saccharides and fatty acids. Table S8: The best hit homologous gene clusters pertaining to all the Red Sea brine SMGCs detected in the study by antiSMASH excluding cf_saccharides and cf_fatty_acids in all the sites included in the Red Sea dataset as computed by ClusterBlast algorithm of antiSMASH [1]. The homologous known gene clusters were detected are also denoted, as well as homologous subclusters. Blue: SMGCs identified in 11/2017 upon re-running the contigs with hits that did not appear in 8/2015. Green: SMGCs identified before in 8/2015 but not when re-run in 11/2017. Table S9: Table showing the percentage of each SMGC as compared to the total SMGCs detected per site among the Red Sea brine samples. Table S10: All the detected archaeal and bacterial genera in all sites included in the dataset.
