# Supplementary material for: Insights into Red Sea Brine Pool Specialized Metabolism Gene Clusters Encoding Potential Metabolites for Biotechnological Applications and Extremophile Survival
Source: Mar Drugs. 2019 May 8;17(5):273. doi: 10.3390/md17050273 (PMC6562949; doi:10.3390/md17050273)
Supplement: Supplementary file 1 [file marinedrugs-17-00273-s001.pdf]

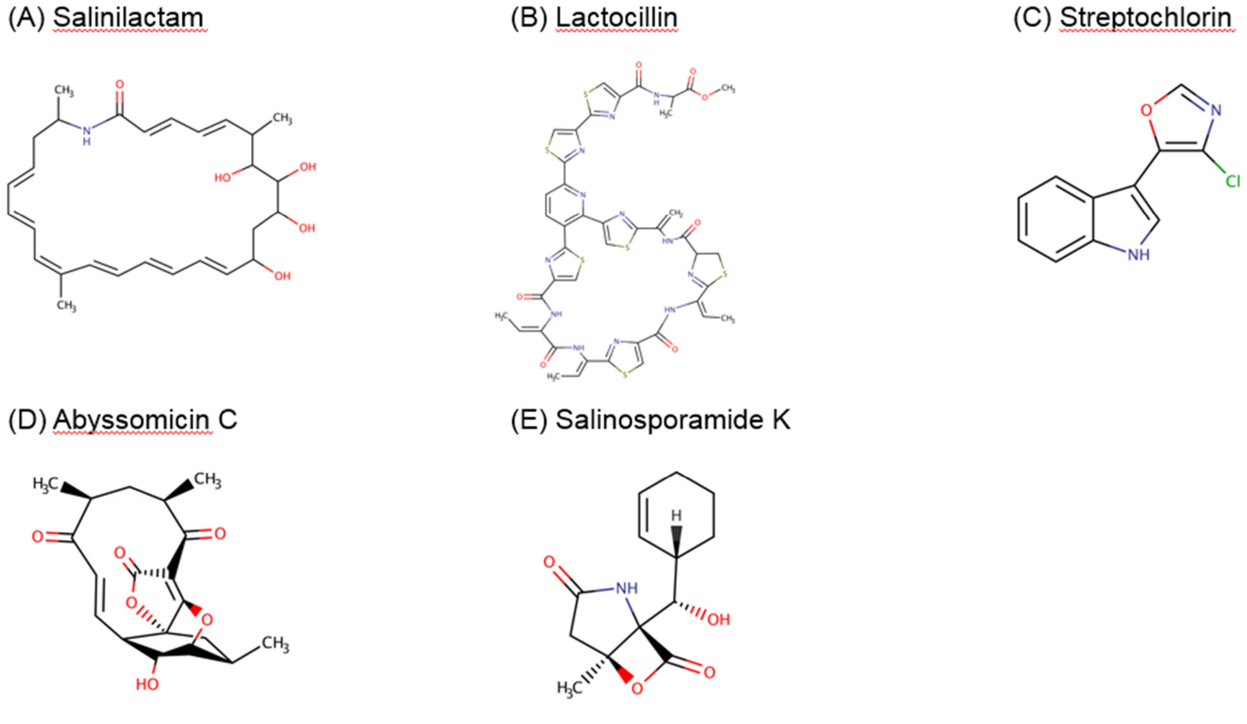

Supplementary figure S1: Chemical structures of some examples of earlier characterized antibiotic and anticancer specialized metabolites: (A) salinilactam, (B) lactocillin, (C) streptochlorin, (D) abyssomicin C and (E) salinosporamide K.

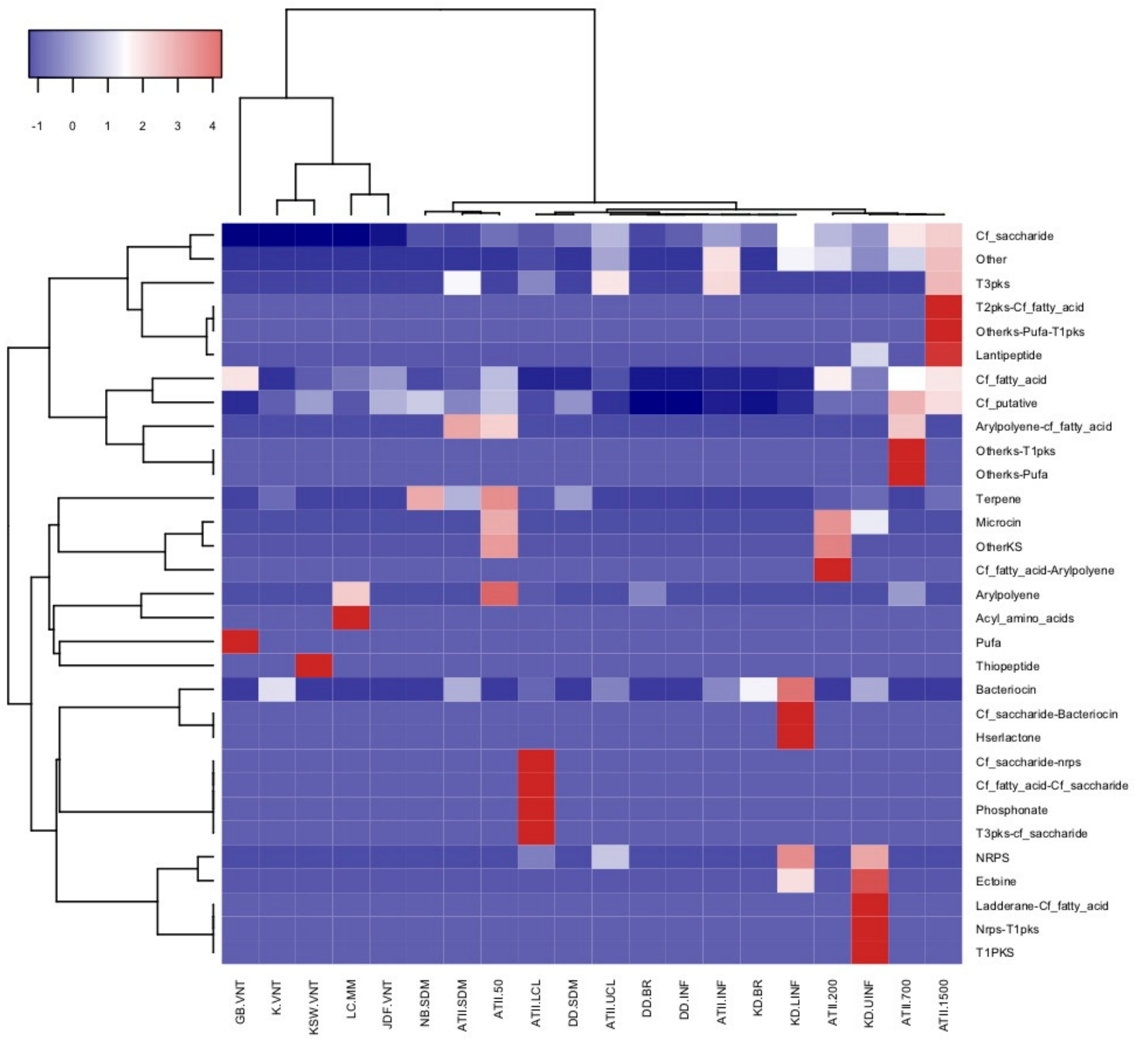

Figure S2. Heat map representing hierarchical classification of the SMGCs detected in all the metagenomes in the dataset.

Table S1: The sampling locations of each of the sites in the dataset.

Samples	Latitude	Longitude	Site depth (m)	Site description	Sample	Sample accession number in SRA	Bio-project accession number in SRA
Atlantis II water column<br><br>(ATII 50, ATII 200, ATII 700, ATII 1500)	21°36'19.0" N	38°12'09.0 "E	50, 200, 700 and 1500	Water column above the brine pool water layers	AT0050m01B1-4C1	SRS598124	PRJNA193416
					AT0200m01C1-4D1	SRS598125	
					AT0700m01C1-3D1	SRS598128	
					AT1500m01B1-3C1	SRS598129	
Atlantis II brine<br><br>(ATII UCL, ATII INF, ATII LCL)	21°36'19.0" N	38°12'09.0 "E	1996–2025	Brine pool water layers	ATBRUCL	SRS1029632	PRJNA193416
					ATBRLCL1-3	SRS1029579	
					ATBRINP	SRS481323	PRJNA219363
Atlantis II - sediments (ATII SDM)	21°36'19.0" N	38°12'09.0 "E	2168 + ~3.5 core length	Sea sediments underlying ATII brine pool	ATIID-1a	SRS1120041	PRJNA299097
					ATIID-1b	SRS1120130	
					ATIID-2	SRS1120133	
					ATIID-3	SRS1120134	
					ATIID-4	SRS1120135	
					ATIID-5	SRS1120142	
					ATIID-6	SRS1120143	

Discovery Deep brine (DD INF, DD BR)	21°17'11.0" N	38°17'14.0 "E	2026–2042	Brine pool water layers	DDBRINP	SRS481325	PRJNA219363
					DDBRINE		
Discovery Deep sediments (DD SDM)	21°17'11.0" N	38°17'14.0 "E	2180 + ~3.5 core length	Sea sediments underlying DD brine pool	DD-1	SRS1120158	PRJNA299097
					DD-2	SRS1120203	
					DD-3	SRS1120205	
					DD-4	SRS1120206	
					DD-5	SRS1120207	
					DD-6	SRS1120208	
					DD-7	SRS1120209	
Kebrit Deep brine (KDBRUINP, KDBRINE, KDBRLINP)	24°43'07.3" N	36°17'19.7 "E	1468-1469	Brine pool water layers	KDBRUINP	SRS481335	PRJNA219363
					KDBRLINP	SRS481337	
					KDBRINE	SRP150921	PRJNA476873
Non-brine sediments (NB SDM)	21°24'31.9" N	38°05'37.4 "E	1856 + 0.37 multi-corer length	Sea sediments underlying brine-influenced site	BI (NBI)	SRS1120214	PRJNA219363
	21°18'09.3" N	38°05'00.2 "E	1937 + ~3.5 core length	Sea sediments underlying brine-influenced site	CD (NBII)	SRS1120210	PRJNA219363
Guaymas Basin deep-sea hydrothermal vent plume water (GB VNT)	27°1.852	111°24.000	1775	Guaymas Basin deep-sea hydrothermal vent plume water	GB VNT	SRS289833	PRJNA77837

Kueishantao shallow-sea hydrothermal vent (water above vent)<br><br>(KSW VNT)	24°50′N	121°57′E	0	G2 (surface water immediately over the vent) water sample from Kueishantao shallow-sea hydrothermal system.	KSW VNT	SRS373507	PRJNA178514
Kueishantao shallow-sea hydrothermal vent (water)<br><br>(K VNT)	24°50′N	121°57′E	17.2	G1 (vent) water sample from Kueishantao shallow-sea hydrothermal system.	K VNT	SRS373506	PRJNA178514
Juan de Fuca Ridge hydrothermal vent diffuse flow seawater<br><br>(JDF VNT)	47°57.00′ N	129°5.81′ W	2198	Juan de Fuca Ridge hydrothermal vent diffuse flow seawater	JDF VNT	SRS172788	PRJNA47471
Loki's Castle deep-sea vent biofilm (microbial mat)<br><br>(LC MM)	73°33.97′N	08°09.51′E	2357	Biofilm (microbial mat) located on the midsection of the surface of black smoker chimney João, in Loki's Castle vent field	LC MM	SRS745051	PRJNA266758

Table S2: The SMGCs detected at each site (absolute values) with the name of the SMGCs detected, the count and total numbers for each site and for each SMGC are denoted.

**Sites**	**ATII SDM**	**DD SDM**	**NB SDM**	**ATII 50**	**ATII 200**	**ATII 700**	**ATII 1500**	**DD BR**	**DD INF**	**ATII INF**	**ATII UCL**	**ATII LCL**	**KD BR**	**KD UINF**	**KD LINF**	**GB VNT**	**KSW VNT**	**K VNT**	**JDF VNT**	**LC MM**	**Total**
**Acyl_amino_acids**	0	0	0	0	0	0	0	0	0	0	0	0	0	0	0	0	0	0	0	1	1

**Arylpolyene**	0	0	0	5	0	1	0	1	0	0	0	1	0	0	0	0	0	0	0	3	11
**Arylpolyene - cf_fatty_acid**	1	0	0	1	0	1	0	0	0	0	0	0	0	0	0	0	0	0	0	0	3
**Bacteriocin**	1	0	0	0	0	0	0	0	0	1	1	3	3	2	6	0	0	1	0	0	18
**Cf_fatty_acid**	7	2	1	21	29	28	18	0	0	2	10	11	2	22	3	9	3	2	1	11	182
**Cf_fatty_acid –Arylpolyene**	0	0	0	0	1	0	0	0	0	0	0	0	0	0	0	0	0	0	0	0	1
**Cf_fatty_acid-Cf_saccharide**	0	0	0	0	0	0	0	0	0	0	0	1	0	0	0	0	0	0	0	0	1
**Cf_putative**	10	14	3	18	9	31	15	0	0	3	6	45	2	17	6	1	5	5	1	7	198
**Cf_saccharide**	46	94	10	85	127	205	129	72	94	152	189	449	142	196	309	0	2	1	1	4	2307
**Cf_saccharide-Bacteriocin**	0	0	0	0	0	0	0	0	0	0	0	0	0	0	3	0	0	0	0	0	3
**Cf_saccharide-nrps**	0	0	0	0	0	0	0	0	0	0	0	1	0	0	0	0	0	0	0	0	1
**Ectoine**	0	0	0	0	0	0	0	0	0	0	0	0	0	2	1	0	0	0	0	0	3
**Hserlactone**	0	0	0	0	0	0	0	0	0	0	0	0	0	0	1	0	0	0	0	0	1
**Ladderane-Cf_fatty_acid**	0	0	0	0	0	0	0	0	0	0	0	0	0	2	0	0	0	0	0	0	2
**Lantipeptide**	0	0	0	0	0	0	1	0	0	0	0	0	0	1	0	0	0	0	0	0	2
**Microcin**	0	0	0	1	1	0	0	0	0	0	0	0	0	1	0	0	0	0	0	0	3
**NRPS**	0	0	0	0	0	0	0	0	0	0	1	2	0	3	3	0	0	0	0	0	9
**Nrps-T1pks**	0	0	0	0	0	0	0	0	0	0	0	0	0	1	0	0	0	0	0	0	1
**Other**	0	0	0	0	1	1	1	0	0	2	1	1	0	1	2	0	0	0	0	0	10
**OtherKS**	0	0	0	1	1	0	0	0	0	0	0	0	0	0	0	0	0	0	0	0	2
**Otherks-Pufa**	0	0	0	0	0	1	0	0	0	0	0	0	0	0	0	0	0	0	0	0	1
**Otherks-Pufa-T1pks**	0	0	0	0	0	0	1	0	0	0	0	0	0	0	0	0	0	0	0	0	1
**Otherks-T1pks**	0	0	0	0	0	1	0	0	0	0	0	0	0	0	0	0	0	0	0	0	1
**Phosphonate**	0	0	0	0	0	0	0	0	0	0	0	1	0	0	0	0	0	0	0	0	1
**Pufa**	0	0	0	0	0	0	0	0	0	0	0	0	0	0	0	1	0	0	0	0	
**T1PKS**	0	0	0	0	0	0	0	0	0	0	0	0	0	1	0	0	0	0	0	0	1
**T2pks-Cf_fatty_acid**	0	0	0	0	0	0	1	0	0	0	0	0	0	0	0	0	0	0	0	0	1
**T3pks**	1	0	0	0	0	0	1	0	0	2	2	3	0	0	0	0	0	0	0	0	9
**T3pks-cf_saccharide**	0	0	0	0	0	0	0	0	0	0	0	1	0	0	0	0	0	0	0	0	1
**Terpene**	4	4	2	14	1	0	1	0	0	0	0	5	0	3	0	0	0	1	0	0	35
**Thiopeptide**	0	0	0	0	0	0	0	0	0	0	0	0	0	0	0	0	1	0	0	0	1
**Total**	70	114	16	146	170	269	168	73	94	162	210	524	149	252	334	11	11	10	3	26	2811

Table S3: The normalized number of SMGCs detected at each site (normalized: number of SMGCs detected/ number of assembled reads at each site $*10^6$), with the name of the SMGCs detected, the count and total numbers for each site and for each SMGC are denoted. In dark blue are the cells with unique SMGCs –only detected once in all the data. Similarly colored cells indicate the SMGCs common to groups of sites (light pink: ATII water column depths, light blue: sediments, yellow: ATII brine layers, bright pink: KD brine layers).

Sites	ATII 50	ATII 200	ATII 700	ATII 1500	ATII SDM	DD SDM	NB SDM	DD BR	DD INF	ATII INF	ATII UCL	ATII LCL	KD BR	KD LINF	KD UINF	GB VNT	KSW VNT	K VNT	JDF VNT	LC MM	Total
**Acyl_amino_acids**	0.00	0.00	0.00	0.00	0.00	0.00	0.00	0.00	0.00	0.00	0.00	0.00	0.00	0.00	0.00	0.00	0.00	0.00	0.00	1.90	1.90
**Arylpolyene**	8.58	0.00	1.80	0.00	0.00	0.00	0.00	1.31	0.00	0.00	0.00	0.26	0.00	0.00	0.00	0.00	0.00	0.00	0.00	5.71	17.66
**Arylpolyene-cf_fatty_acid**	1.72	0.00	1.80	0.00	2.09	0.00	0.00	0.00	0.00	0.00	0.00	0.00	0.00	0.00	0.00	0.00	0.00	0.00	0.00	0.00	5.61
**Bacteriocin**	0.00	0.00	0.00	0.00	2.09	0.00	0.00	0.00	0.00	1.35	1.26	0.77	3.28	6.48	1.96	0.00	0.00	2.95	0.00	0.00	20.14
**Cf_fatty_acid**	36.03	54.67	50.51	56.94	14.63	3.35	10.81	0.00	0.00	2.69	12.58	2.82	2.19	3.24	21.55	57.75	15.06	5.91	28.28	20.92	399.94
**Cf_fatty_acid-Arylpolyene**	0.00	1.89	0.00	0.00	0.00	0.00	0.00	0.00	0.00	0.00	0.00	0.00	0.00	0.00	0.00	0.00	0.00	0.00	0.00	0.00	1.89
**Cf_fatty_acid-Cf_saccharide**	0.00	0.00	0.00	0.00	0.00	0.00	0.00	0.00	0.00	0.00	0.00	0.26	0.00	0.00	0.00	0.00	0.00	0.00	0.00	0.00	0.26
**Cf_putative**	30.89	16.97	55.92	47.45	20.90	23.43	32.42	0.00	0.00	4.04	7.55	11.53	2.19	6.48	16.65	6.42	25.10	14.77	28.28	13.32	364.30
**Cf_saccharide**	145.86	239.42	369.81	408.10	96.14	157.31	108.07	94.32	125.00	204.56	237.82	115.07	155.39	333.57	192.02	0.00	10.04	2.95	28.28	7.61	3031.34
**Cf_saccharide-Bacteriocin**	0.00	0.00	0.00	0.00	0.00	0.00	0.00	0.00	0.00	0.00	0.00	0.00	0.00	3.24	0.00	0.00	0.00	0.00	0.00	0.00	3.24
**Cf_saccharide-nrps**	0.00	0.00	0.00	0.00	0.00	0.00	0.00	0.00	0.00	0.00	0.00	0.26	0.00	0.00	0.00	0.00	0.00	0.00	0.00	0.00	0.26
**Ectoine**	0.00	0.00	0.00	0.00	0.00	0.00	0.00	0.00	0.00	0.00	0.00	0.00	0.00	1.08	1.96	0.00	0.00	0.00	0.00	0.00	3.04
**Hserlactone**	0.00	0.00	0.00	0.00	0.00	0.00	0.00	0.00	0.00	0.00	0.00	0.00	0.00	1.08	0.00	0.00	0.00	0.00	0.00	0.00	1.08
**Ladderane-Cf_fatty_acid**	0.00	0.00	0.00	0.00	0.00	0.00	0.00	0.00	0.00	0.00	0.00	0.00	0.00	0.00	1.96	0.00	0.00	0.00	0.00	0.00	1.96
**Lantipeptide**	0.00	0.00	0.00	3.16	0.00	0.00	0.00	0.00	0.00	0.00	0.00	0.00	0.00	0.00	0.98	0.00	0.00	0.00	0.00	0.00	4.14
**Microcin**	1.72	1.89	0.00	0.00	0.00	0.00	0.00	0.00	0.00	0.00	0.00	0.00	0.00	0.00	0.98	0.00	0.00	0.00	0.00	0.00	4.58
**NRPS**	0.00	0.00	0.00	0.00	0.00	0.00	0.00	0.00	0.00	0.00	1.26	0.51	0.00	3.24	2.94	0.00	0.00	0.00	0.00	0.00	7.95
**Nrps-T1pks**	0.00	0.00	0.00	0.00	0.00	0.00	0.00	0.00	0.00	0.00	0.00	0.00	0.00	0.00	0.98	0.00	0.00	0.00	0.00	0.00	0.98

**Other**	0.00	1.89	1.80	3.16	0.00	0.00	0.00	0.00	0.00	2.69	1.26	0.26	0.00	2.16	0.98	0.00	0.00	0.00	0.00	0.00	14.20
**OtherKS**	1.72	1.89	0.00	0.00	0.00	0.00	0.00	0.00	0.00	0.00	0.00	0.00	0.00	0.00	0.00	0.00	0.00	0.00	0.00	0.00	3.60
**Otherks-Pufa**	0.00	0.00	1.80	0.00	0.00	0.00	0.00	0.00	0.00	0.00	0.00	0.00	0.00	0.00	0.00	0.00	0.00	0.00	0.00	0.00	1.80
**Otherks-Pufa-T1pks**	0.00	0.00	0.00	3.16	0.00	0.00	0.00	0.00	0.00	0.00	0.00	0.00	0.00	0.00	0.00	0.00	0.00	0.00	0.00	0.00	3.16
**Otherks-T1pks**	0.00	0.00	1.80	0.00	0.00	0.00	0.00	0.00	0.00	0.00	0.00	0.00	0.00	0.00	0.00	0.00	0.00	0.00	0.00	0.00	1.80
**Phosphonate**	0.00	0.00	0.00	0.00	0.00	0.00	0.00	0.00	0.00	0.00	0.00	0.26	0.00	0.00	0.00	0.00	0.00	0.00	0.00	0.00	0.26
**Pufa**	0.00	0.00	0.00	0.00	0.00	0.00	0.00	0.00	0.00	0.00	0.00	0.00	0.00	0.00	0.00	6.42	0.00	0.00	0.00	0.00	6.42
**T1PKS**	0.00	0.00	0.00	0.00	0.00	0.00	0.00	0.00	0.00	0.00	0.00	0.00	0.00	0.00	0.98	0.00	0.00	0.00	0.00	0.00	0.98
**T2pks-Cf_fatty_acid**	0.00	0.00	0.00	3.16	0.00	0.00	0.00	0.00	0.00	0.00	0.00	0.00	0.00	0.00	0.00	0.00	0.00	0.00	0.00	0.00	3.16
**T3pks**	0.00	0.00	0.00	3.16	2.09	0.00	0.00	0.00	0.00	2.69	2.52	0.77	0.00	0.00	0.00	0.00	0.00	0.00	0.00	0.00	11.23
**T3pks-cf_saccharide**	0.00	0.00	0.00	0.00	0.00	0.00	0.00	0.00	0.00	0.00	0.00	0.26	0.00	0.00	0.00	0.00	0.00	0.00	0.00	0.00	0.26
**Terpene**	24.02	1.89	0.00	3.16	8.36	6.69	21.61	0.00	0.00	0.00	0.00	1.28	0.00	0.00	2.94	0.00	0.00	2.95	0.00	0.00	72.92
**Thiopeptide**	0.00	0.00	0.00	0.00	0.00	0.00	0.00	0.00	0.00	0.00	0.00	0.00	0.00	0.00	0.00	0.00	5.02	0.00	0.00	0.00	5.02
**Total**	250.53	320.49	485.27	531.48	146.30	190.78	172.92	95.63	125.00	218.02	264.25	134.29	163.05	360.56	246.88	70.58	55.21	29.54	84.85	49.46	3990.05

Table S4: Archaeal and Bacterial and viral phyla detected for each of the Red Sea brine assembly files. The relative abundance is shown whenever detected to be ≥ 0.5% in at least one of the assemblies.

	ATII SDM	DD SDM	NB SDM	DD BR	DD INF	ATII 50	ATII 200	ATII 700	ATII 1500	ATII INF	ATII LCL	ATII UCL	KD BR	KD LINF	KD UINF
**Archaea**	6.84	0.51	0.00	28.15	24.42	0.14	30.18	34.28	32.59	0.84	4.50	0.82	58.15	73.30	28.06
**Crenarchaeota**	0.05	0.01	0.00	0.14	0.39	0.00	0.27	0.27	0.25	0.07	0.04	0.05	2.43	0.52	0.13
**Euryarchaeota**	0.26	0.12	0.00	6.51	6.59	0.12	0.78	0.93	1.22	0.73	0.78	0.61	54.59	6.01	1.25
**Korarchaeota**	0.00	0.00	0.00	0.00	0.00	0.00	0.00	0.00	0.01	0.00	0.00	0.00	0.75	0.07	0.00
**Thaumarchaeota**	6.53	0.38	0.00	21.50	17.44	0.02	29.12	33.08	31.09	0.04	3.68	0.15	0.09	66.62	26.68
**Bacteria**	59.03	46.41	52.17	67.75	71.90	97.02	68.27	64.80	65.88	98.58	95.05	98.83	39.89	26.05	71.30
**Acidobacteria**	0.30	0.40	0.40	0.42	0.78	0.08	0.38	0.51	0.53	0.02	0.08	0.06	0.28	0.19	0.82
**Actinobacteria**	0.21	0.26	0.09	0.85	1.16	0.70	2.16	1.87	1.09	0.75	0.64	0.65	1.31	0.83	3.34
**Aquificae**	0.02	0.01	0.00	0.00	0.00	0.03	0.14	0.17	0.24	0.00	0.03	0.03	1.59	0.58	0.38
**Bacteroidetes**	6.46	9.13	8.16	1.56	2.71	6.66	4.44	3.65	4.39	0.09	0.38	0.24	2.81	1.67	4.50
**Candidatus Poribacteria**	0.08	0.00	0.00	2.97	3.10	0.00	0.50	1.07	0.83	0.00	0.00	0.00	0.00	0.07	0.18
**Chlorobi**	0.06	0.01	0.02	0.42	0.78	0.13	0.85	0.93	1.33	0.04	0.31	0.17	0.66	0.78	2.99
**Chloroflexi**	0.11	0.01	0.00	1.70	0.00	0.17	0.60	0.66	1.06	0.04	0.09	0.03	0.66	1.15	1.02
**Cyanobacteria**	19.46	25.80	38.18	0.42	1.16	29.97	0.82	0.58	0.93	0.02	0.19	0.12	0.66	0.78	1.53
**Firmicutes**	0.37	0.38	0.13	4.24	3.49	0.59	1.91	2.17	2.74	0.11	0.47	0.23	16.57	8.20	6.06
**Nitrospirae**	0.07	0.00	0.00	2.26	3.29	0.03	0.79	1.53	1.20	0.00	0.05	0.04	0.47	0.38	0.74
**Planctomycetes**	0.07	0.04	0.00	0.42	0.58	0.15	0.47	0.54	0.65	0.02	0.09	0.03	0.00	0.15	1.12
**Proteobacteria**	31.51	10.23	5.12	51.63	52.33	57.97	53.41	49.44	48.86	97.16	92.44	97.03	9.18	7.07	45.04
**Spirochaetes**	0.09	0.02	0.05	0.14	0.00	0.08	0.37	0.28	0.33	0.00	0.03	0.01	0.00	0.43	0.53
**Synergistetes**	0.00	0.00	0.00	0.00	0.00	0.01	0.11	0.06	0.12	0.02	0.01	0.00	0.66	0.24	0.21
**Thermotogae**	0.01	0.00	0.00	0.14	0.58	0.07	0.13	0.08	0.14	0.13	0.07	0.10	3.28	1.02	0.36
**Verrucomicrobia**	0.04	0.02	0.00	0.14	0.39	0.09	0.38	0.41	0.47	0.00	0.04	0.03	0.00	0.20	0.60
**unclassified (derived from Bacteria)**	0.01	0.01	0.00	0.14	0.00	0.03	0.06	0.08	0.16	0.04	0.03	0.00	0.00	0.83	0.49
**Viruses**															
**unclassified (derived from Viruses)**	33.81	52.72	47.46	3.96	3.49	2.54	0.84	0.42	0.71	0.49	0.34	0.23	1.87	0.30	0.28

Table S5: All the detected archaeal and bacterial phyla in all the Red Sea brine sites by MG-RAST. The relative abundance is shown for all the phyla and values > 0 are highlighted in red.

	ATII SDM	DD SDM	NB SDM	DD BR	DD INF	ATII 50	ATII 200	ATII 700	ATII 1500	ATII INF	ATII LCL	ATII UCL	KD BR	KD LINF	KD UINF
Acidobacteria	0.305	0.399	0.397	0.424	0.775	0.081	0.378	0.510	0.529	0.020	0.080	0.060	0.281	0.185	0.816
Actinobacteria	0.211	0.261	0.090	0.849	1.163	0.702	2.163	1.869	1.092	0.755	0.636	0.653	1.311	0.834	3.342
Aquificae	0.020	0.010	0.000	0.000	0.000	0.030	0.141	0.169	0.240	0.000	0.030	0.030	1.592	0.579	0.383

Bacteroidetes	6.463	9.129	8.156	1.556	2.713	6.656	4.437	3.648	4.394	0.090	0.380	0.243	2.809	1.668	4.499
Candidatus Poribacteria	0.080	0.000	0.000	2.970	3.101	0.000	0.496	1.071	0.831	0.000	0.000	0.000	0.000	0.070	0.179
Chlamydiae	0.044	0.030	0.000	0.000	0.194	0.029	0.069	0.075	0.110	0.000	0.000	0.000	0.187	0.093	0.060
Chlorobi	0.062	0.010	0.020	0.424	0.775	0.134	0.851	0.927	1.325	0.044	0.311	0.167	0.655	0.776	2.993
Chloroflexi	0.112	0.010	0.000	1.697	0.000	0.166	0.595	0.658	1.057	0.044	0.090	0.030	0.655	1.147	1.020
Chrysiogenetes	0.010	0.000	0.000	0.000	0.388	0.014	0.038	0.011	0.048	0.000	0.000	0.000	0.281	0.081	0.111
Crenarchaeota	0.050	0.010	0.000	0.141	0.388	0.004	0.275	0.266	0.254	0.067	0.040	0.050	2.434	0.521	0.128
Cyanobacteria	19.464	25.804	38.181	0.424	1.163	29.970	0.824	0.579	0.927	0.022	0.190	0.115	0.655	0.776	1.531
Deferribacteres	0.020	0.000	0.020	0.000	0.000	0.052	0.084	0.108	0.144	0.000	0.014	0.000	0.187	0.174	0.366
Deinococcus-Thermus	0.020	0.010	0.000	0.141	0.388	0.091	0.168	0.162	0.185	0.020	0.046	0.013	0.281	0.197	0.264
Dictyoglomi	0.020	0.000	0.000	0.000	0.388	0.014	0.042	0.018	0.021	0.044	0.005	0.026	0.468	0.301	0.068
Elusimicrobia	0.000	0.000	0.000	0.000	0.000	0.006	0.023	0.014	0.021	0.000	0.005	0.013	0.000	0.174	0.094
Euryarchaeota	0.261	0.118	0.000	6.506	6.589	0.118	0.782	0.934	1.222	0.732	0.784	0.615	54.588	6.013	1.250
Fibrobacteres	0.000	0.000	0.000	0.000	0.000	0.002	0.011	0.032	0.007	0.000	0.000	0.000	0.000	0.035	0.026
Firmicutes	0.373	0.379	0.126	4.243	3.488	0.594	1.912	2.174	2.739	0.111	0.473	0.231	16.573	8.202	6.063
Fusobacteria	0.000	0.010	0.000	0.141	0.194	0.017	0.046	0.022	0.034	0.000	0.000	0.000	0.375	0.232	0.162
Gemmatimonadetes	0.000	0.000	0.000	0.000	0.000	0.004	0.221	0.244	0.199	0.022	0.010	0.000	0.000	0.035	0.060
Korarchaeota	0.000	0.000	0.000	0.000	0.000	0.000	0.000	0.000	0.014	0.000	0.000	0.000	0.749	0.070	0.000
Lentisphaerae	0.010	0.000	0.000	0.000	0.000	0.010	0.050	0.075	0.014	0.022	0.010	0.026	0.000	0.023	0.111
Nanoarchaeota	0.000	0.000	0.000	0.000	0.000	0.000	0.000	0.004	0.007	0.000	0.000	0.000	0.281	0.070	0.010
Nitrospirae	0.070	0.000	0.000	2.263	3.295	0.030	0.786	1.535	1.201	0.000	0.046	0.038	0.468	0.382	0.740
Planctomycetes	0.075	0.040	0.000	0.424	0.581	0.155	0.465	0.543	0.652	0.020	0.088	0.030	0.000	0.151	1.123
Proteobacteria	31.507	10.227	5.125	51.627	52.326	57.970	53.413	49.438	48.860	97.159	92.438	97.028	9.176	7.067	45.038
Spirochaetes	0.090	0.020	0.050	0.141	0.000	0.080	0.374	0.280	0.330	0.000	0.028	0.010	0.000	0.429	0.527
Synergistetes	0.000	0.000	0.000	0.000	0.000	0.010	0.107	0.061	0.124	0.020	0.014	0.000	0.655	0.243	0.213
Tenericutes	0.020	0.000	0.000	0.000	0.000	0.014	0.000	0.011	0.021	0.000	0.000	0.000	0.000	0.151	0.060
Thaumarchaeota	6.525	0.384	0.000	21.499	17.442	0.017	29.124	33.077	31.093	0.040	3.679	0.154	0.094	66.624	26.677
Thermotogae	0.010	0.000	0.000	0.141	0.581	0.070	0.134	0.075	0.144	0.133	0.070	0.102	3.277	1.019	0.357
unclassified (derived from Bacteria)	0.010	0.010	0.000	0.141	0.000	0.030	0.065	0.079	0.158	0.040	0.030	0.000	0.000	0.834	0.493
Verrucomicrobia	0.040	0.020	0.000	0.141	0.388	0.090	0.378	0.413	0.474	0.000	0.040	0.030	0.000	0.197	0.604

Table S6: The most abundant archaeal and bacterial genera in all the sites in the dataset.

Site	The most abundant archaeal genus (% relative abundance)	The most abundant bacterial genus (% relative abundance)
**ATII 50**	*Methanococcus* (0.02 %)	*Candidatus Pelagibacter* (44.25 %)
**ATII 200**	*Nitrosopumilus* (25.06 %)	*Candidatus Pelagibacter* (26.17 %)
**ATII 700**	*Nitrosopumilus* (28.85 %)	*Candidatus Pelagibacter* (29.67 %)
**ATII 1500**	*Nitrosopumilus* (27.09 %)	*Candidatus Pelagibacter* (27.12 %)
**ATII INF**	*Halorhabdus* (0.53 %)	*Cupriavidus* (14.98 %)
**ATII UCL**	*Halorhabdus* (0.41 %)	*Cupriavidus* (17.48 %)
**ATII LCL**	*Nitrosopumilus* (3.52 %)	*Cupriavidus* (30.25 %)
**DD INF**	*Nitrosopumilus* (15.50 %)	*Candidatus Pelagibacter* (19.38 %)
**DD BR**	*Nitrosopumilus* (18.81 %)	*Candidatus Pelagibacter* (23.34 %)
**KD UINF**	*Nitrosopumilus* (25.67 %)	*Mariprofundus* (8.05 %)
**KD LINF**	*Nitrosopumilus* (64.59 %)	*Clostridium* (1.29 %)
**KD BR**	*Thermococcus* (5.62 %)	*Clostridium* (2.15 %)
**ATII SDM**	*Nitrosopumilus* (5.67 %)	*Prochlorococcus* (18.24 %)
**DD SDM**	*Nitrosopumilus* (0.35 %)	*Prochlorococcus* (23.93 %)
**NB SDM**	-	*Prochlorococcus* (36.77 %)
**GB VNT**	*Nitrosopumilus* (26.65 %)	*unclassified (derived from Gammaproteobacteria)* (18.87 %)
**KSW VNT**	*Thermococcus* (9.85 %)	*Nautilia* (40.49 %)

**K VNT**	*Methanococcoides* (0.044 %)	*Candidatus Pelagibacter* (56.88 %)
**JDF VNT**	*Thermococcus* (15.41 %)	*Nautilia* (28.87 %)
**LC MM**	*Aciduliprofundum* (0.03 %)	*Sulfurovum* (40.54 %)

Table S7: Table showing the rare leucine TTA codon absolute and normalized count in each site, for all the Red Sea brine SMGCs excluding the saccharides and fatty acids.

Sites	Count of TTA codons in all SMGCs excluding saccharides and fatty acids	Number of assembled reads	Normalized number of TTA codons *106
**ATII 50**	**1267**	**582,768**	**2174**
**ATII 200**	**317**	**530,441**	**598**
**ATII 700**	**1459**	**554,335**	**2632**
**ATII 1500**	**527**	**316,101**	**1667**
**ATII INF**	**45**	**743,064**	**61**
**ATII UCL**	**77**	**794,715**	**97**
**ATII LCL**	**388**	**3,901,967**	**99**
**DD INF**	**0**	**752,025**	**0**
**DD BR**	**12**	**763,387**	**16**
**KD UINF**	**1305**	**1,020,749**	**1278**
**KD LINF**	**649**	**926,337**	**701**
**KD BR**	**66**	**913,803**	**72**
**ATII SDM**	**745**	**478,453**	**1557**
**DD SDM**	**721**	**597,552**	**1207**
**NB SDM**	**106**	**92,530**	**1146**

**Total**	**7,684**	**12,968,227**	**13,305**

Table S8: The best hit homologous gene clusters pertaining to all the Red Sea brine SMGCs detected in the study by antiSMASH excluding cf_saccharides and cf_fatty_acids in all the sites included in the Red Sea dataset as computed by ClusterBlast algorithm of antiSMASH [1]. The homologous known gene clusters were detected are also denoted, as well as homologous subclusters. Blue: SMGCs identified in 11/2017 upon re-running the contigs with hits that did not appear in 8/2015. Green: SMGCs identified before in 8/2015 but not when re-run in 11/2017.

**Site**	**Contig name**	**Cluster antiSMASH output**	**Homologous gene cluster best hit (ClusterBlast) by antiSMASH**
	contig00013length=14769numreads=255	cf_putative	LDPY01000001_c2: Puniceibacterium sp. IMCC21224 IMCC21224 11, whole genome ... (8% of genes show similarity).
	contig00019length=13554numreads=265	cf_putative	DS999411_c1: Luminiphilus syltensis NOR51-B scf 1109846220923 genomic scaff... (6% of genes show similarity).
	contig00044length=10189numreads=133	cf_putative	No significant ClusterBlast hits found.
	contig00047length=10108numreads=209	cf_putative	LAXI01000011_c2: Roseovarius indicus strain B108 contig11, whole genome sho... (10% of genes show similarity) and **Homologous known gene cluster: BGC0000867: Polyhydroxyalkanoic acids biosynthetic gene cluster (28% of genes show similarity).**
	contig00077length=8217numreads=125	cf_putative	ADNU01000022_c1: Brevibacterium mcbrellneri ATCC 49030 contig00024, whole g... (5% of genes show similarity).
**ATII 50**	contig00083length=8026numreads=135	cf_putative	JNBY01000073_c1: Kitasatospora cheerisanensis KCTC 2395 contig00073, whole ... (3% of genes show similarity).

	contig00084length=8024numreads=145	cf_putative	LN827929_c2: Candidatus Methylopumilus planktonicus genome assembly Candida... (5% of genes show similarity).
	contig00129length=7083numreads=112	cf_putative	JHDT01000005_c1: Planococcus sp. L10.15 contig6, whole genome shotgun seque... (8% of genes show similarity).
	contig00153length=6726numreads=64	cf_putative	AGFR01000009_c1: Commensalibacter intestini A911 74 9, whole genome shotgun... (4% of genes show similarity).
	contig00320length=4988numreads=69	cf_putative	LN813019_c3: Halomonas sp. R57-5 genome assembly HalomonasR57-5, chromosome... (7% of genes show similarity).
	contig00331length=4887numreads=96	cf_putative	CP009144_c6: Sinorhizobium meliloti strain RMO17, complete genome. (4% of genes show similarity).
	contig00399length=4463numreads=46	cf_putative	No significant ClusterBlast hits found.
	contig00413length=4451numreads=60	cf_putative	JJOB01000001_c8: Streptomyces sp. NTK 937 contig00001, whole genome shotgun... (8% of genes show similarity).
	contig00520length=4046numreads=70	cf_putative	LDPO01000007_c3: Mycobacterium heraklionense strain Davo contig 7, whole ge... (6% of genes show similarity).
	contig00633length=3760numreads=60	cf_putative	AP013068_c4: Pseudomonas resinovorans NBRC 106553 DNA, complete geonome. (5% of genes show similarity).
	contig01016length=3080numreads=16	cf_putative	ATNM01000147_c1: Cyclobacterium qasimii M12-11B contig00007, whole genome s... (6% of genes show similarity).
	contig01156length=2944numreads=27	cf_putative	No significant ClusterBlast hits found.
	contig01700length=2527numreads=16	cf_putative	AP012305_c1: Azoarcus sp. KH32C plasmid pAZKH DNA, complete genome. (5% of genes show similarity).

	contig00874length=3229numreads=25	Terpene	AAPV01000001_c1: Candidatus Pelagibacter ubique HTCC1002 1099314759167, who... (8% of genes show similarity).
	contig02940length=2067numreads=140	Terpene	CP000576_c3: Prochlorococcus marinus str. MIT 9301, complete genome. (4% of genes show similarity).
	contig04224length=1755numreads=11	Terpene	CP000084_c1: Candidatus Pelagibacter ubique HTCC1062, complete genome. (4% of genes show similarity).
	contig04324length=1774numreads=18	Terpene	CP002511_c1: Candidatus Pelagibacter sp. IMCC9063, complete genome. (7% of genes show similarity).
	contig04568length=1739numreads=10	Terpene	LICT01000042_c1: Pelagibacteraceae bacterium BACL20 MAG-120920-bin64 contig... (33% of genes show similarity).
	contig05278length=1610numreads=14	Terpene	ADAC02000001_c1: Alpha proteobacterium HIMB114 HIMB114 HIMB114, whole genom... (4% of genes show similarity).
	contig07162length=1447numreads=7	Terpene	DS995298_c1: Candidatus Pelagibacter sp. HTCC7211 scf 1105874033148 genomic... (7% of genes show similarity) and **Homologous known gene cluster: BGC0000634: Carotenoid biosynthetic gene cluster (16% of genes show similarity).**
	contig08846length=1328numreads=13	Terpene	No significant ClusterBlast hits found.
	contig12560length=1123numreads=7	Terpene	JNAI01000007_c1: Prochlorococcus marinus str. MIT 9107 contig00005, whole g... (4% of genes show similarity).
	contig07777length=1388numreads=18	Terpene	DS999537_c3: Prochlorococcus marinus str. MIT 9202 scf 1109798535001 genomi... (4% of genes show similarity).
	contig07804length=1390numreads=10	Terpene	AAXX01000001_c1: Flavobacteria bacterium BAL38 1101699887718, whole genome ... (4% of genes show similarity) and **Homologous known gene cluster: BGC0000650: Carotenoid biosynthetic gene cluster (28% of genes show similarity).**

	contig09506length=1266numreads=10	Terpene	
	contig13130length=1083numreads=11	Terpene	No significant ClusterBlast hits found.
	contig13810length=1065numreads=9	Terpene	No significant ClusterBlast hits found.
	contig07660length=1404numreads=9	Arylpolyene - cf_fatty_acid	CP006877_c1: Rhizobium gallicum bv. gallicum R602, complete genome. (4% of genes show similarity).
	contig00927length=3221numreads=59	Microcin	
	contig02297length=2268numreads=25	Arylpolyene	CP006877_c1: Rhizobium gallicum bv. gallicum R602, complete genome. (7% of genes show similarity).
	contig07217length=1446numreads=4	Arylpolyene	CP000282_c2: Saccharophagus degradans 2-40, complete genome. (8% of genes show similarity).
	contig12511length=1112numreads=11	Arylpolyene	No significant ClusterBlast hits found.
	contig08136length=1384numreads=38	Arylpolyene	CP006877_c1: Rhizobium gallicum bv. gallicum R602, complete genome. (4% of genes show similarity) and **Homologous known gene cluster: BGC0000358: Fusaricidin biosynthetic gene cluster (25% of genes show similarity).**
	contig13317length=1095numreads=8	Arylpolyene	CP000282_c2: Saccharophagus degradans 2-40, complete genome. (5% of genes show similarity).
	contig00634length=3707numreads=23	Otherks	AGIZ01000005_c3: Fischerella sp. JSC-11 ctg112, whole genome shotgun sequen... (3% of genes show similarity).
**ATII 200**	contig00023length=7478numreads=67	cf_putative	LJTL01000006_c1: Desulfobacterales bacterium SG8 35 2 WOR 8-12 525, whole g... (9% of genes show similarity).

	contig00127length=4855numreads=89	cf_putative	CP000431_c18: Rhodococcus jostii RHA1, complete genome. (2% of genes show similarity) and **Homologous known gene cluster: AY271660_3_c3: maduropeptin aminosugar (madurose) (40% of genes show similarity).**
	contig00241length=4176numreads=58	cf_putative	CYPU01000022_c1: Ruegeria atlantica genome assembly R.atlanticaCECT4292 Seq... (15% of genes show similarity).
	contig00265length=4091numreads=34	cf_putative	CP001344_c6: Cyanothece sp. PCC 7425, complete genome. (5% of genes show similarity).
	contig00401length=3654numreads=64	cf_putative	CXWD01000030_c1: Labrenzia alexandrii genome assembly L.alexandriiCECT5112 ... (9% of genes show similarity).
	contig00493length=3468numreads=22	cf_putative	FQ311869_c1: Azospirillum lipoferum 4B plasmid AZO p1 complete genome. (12% of genes show similarity).
	contig00603length=3254numreads=25	cf_putative	No significant ClusterBlast hits found.
	contig00754length=3034numreads=43	cf_putative	No significant ClusterBlast hits found.
	contig01799length=2332numreads=11	cf_putative	AYJU01000003_c1: Brevibacillus panacihumi W25 contig 11, whole genome shotg... (5% of genes show similarity).
	contig05461length=1611numreads=4	Terpene	No significant ClusterBlast hits found.
	contig06328length=1524numreads=12	Other	No significant ClusterBlast hits found.
	contig00099length=5113numreads=51	Cf_fatty_acid-Arylpolyene	CP002738_c2: Methylomonas methanica MC09, complete genome. (5% of genes show similarity).
	contig12113length=1157numreads=13	Microcin	No significant ClusterBlast hits found.

	contig10963length=1202numreads=13	Otherks	No significant ClusterBlast hits found.
ATII 700	contig00026length=16426numreads=275	cf_putative	AFWT01000016_c1: Thiorhodococcus drewsii AZ1 ctg196, whole genome shotgun s... (5% of genes show similarity).
	contig00053length=13004numreads=161	cf_putative	LMSN01000019_c1: Nocardioides sp. Soil777 contig 7, whole genome shotgun se... (5% of genes show similarity).
	contig00064length=12445numreads=205	cf_putative	CP001052_c2: Burkholderia phytofirmans PsJN chromosome 1, complete sequence. (5% of genes show similarity).
	contig00071length=12145numreads=163	cf_putative	AAYC01000014_c1: Roseobacter sp. SK209-2-6 1101232001818, whole genome shot... (4% of genes show similarity).
	contig00079length=11906numreads=155	cf_putative	No significant ClusterBlast hits found.
	contig00089length=11623numreads=141	cf_putative	DS989841_c1: Microcoleus chthonoplastes PCC 7420 scf 1103659003829 genomic ... (5% of genes show similarity).
	contig00110length=10537numreads=112	cf_putative	No significant ClusterBlast hits found.
	contig00112length=10487numreads=160	cf_putative	LFEJ01000003_c1: Cronobacter sp. DJ34 Bact A5 1, whole genome shotgun seque... (8% of genes show similarity).
	contig00153length=9327numreads=127	cf_putative	CP006880_c3: Rhizobium gallicum bv. gallicum R602 plasmid pRgalR602c, compl... (4% of genes show similarity).
	contig00205length=7681numreads=129	cf_putative	CP005929_c2: Actinoplanes sp. N902-109, complete genome. (2% of genes show similarity).
	contig00263length=6790numreads=80	cf_putative	LJTV01000005_c1: Gammaproteobacteria bacterium SG8 47 WOR 8-12 900, whole g... (14% of genes show similarity).

	contig00264length=6764numreads=40	cf_putative	BAFN01000001_c3: Candidatus Brocadia sinica JPN1 DNA, contig: brosiA, whole... (4% of genes show similarity).
	contig00317length=6161numreads=59	cf_putative	No significant ClusterBlast hits found.
	contig00359length=5754numreads=73	cf_putative	LADP01000022_c1: Peptococcaceae bacterium BRH c8a BRHa 1002202, whole genom... (12% of genes show similarity).
	contig00693length=4145numreads=53	cf_putative	No significant ClusterBlast hits found.
	contig00697length=4138numreads=37	cf_putative	CP008947_c16: Rhodococcus opacus strain R7 sequence. (4% of genes show similarity).
	contig00726length=4073numreads=24	cf_putative	No significant ClusterBlast hits found.
	contig01098length=3423numreads=31	cf_putative	AFVW02000018_c1: Mycobacterium colombiense CECT 3035 contig00034, whole gen... (8% of genes show similarity).
	contig01159length=3330numreads=25	cf_putative	JTDW01000010_c1: Tamlana sedimentorum strain JCM 19808 contig11, whole geno... (5% of genes show similarity).
	contig01419length=3062numreads=55	cf_putative	CP010904_c3: Verrucomicrobia bacterium L21-Fru-AB, complete genome. (5% of genes show similarity).
	contig01480length=3019numreads=24	cf_putative	GG704596_c4: Silicibacter lacuscaerulensis ITI-1157 genomic scaffold scf 11... (7% of genes show similarity).
	contig01500length=3015numreads=33	cf_putative	
	contig01633length=2887numreads=28	cf_putative	DF970134_c1: Mizugakiibacter sediminis DNA, contig: NODE 4, strain: skMP5, ... (4% of genes show similarity).

	contig01716length=2824numreads=15	cf_putative	No significant ClusterBlast hits found.
	contig02389length=2429numreads=31	cf_putative	
	contig04362length=1887numreads=15	cf_putative	No significant ClusterBlast hits found.
	contig05404length=1723numreads=9	cf_putative	CP000110_c1: Synechococcus sp. CC9605, complete genome. (7% of genes show similarity).
	contig05461length=1672numreads=18	cf_putative	AMBZ01000002_c1: Paenibacillus alvei DSM 29 PAV 2c, whole genome shotgun se... (6% of genes show similarity).
	contig06917length=1536numreads=17	cf_putative	HE971709_c11: Streptomyces davawensis strain JCM 4913 complete genome. (2% of genes show similarity), **Homologous subcluster: AB088119_2_c2: staurosporine deoxysugar (20% of genes show similarity) and Homologous known gene cluster: BGC0000167: Vicenistatin biosynthetic gene cluster (10% of genes show similarity).**
	contig03168length=2152numreads=24	cf_putative	CP010407_c10: Streptomyces vietnamensis strain GIM4.0001, complete genome. (2% of genes show similarity) and **Homologous known gene cluster: BGC0000796: S-layer glycan biosynthetic gene cluster (9% of genes show similarity).**
	contig03253length=2129numreads=6	cf_putative	
	contig00399length=5472numreads=81	Other	No significant ClusterBlast hits found.
	contig02538length=2370numreads=39	Arylpolyene - cf_fatty_acid	CP006877_c1: Rhizobium gallicum bv. gallicum R602, complete genome. (7% of genes show similarity).

	contig00087length=11659numreads=148	Otherks-Pufa	JTCM01000043_c2: Hassallia byssoidea VB512170 scaffold 0, whole genome shot... (13% of genes show similarity).
	contig00145length=9596numreads=132	Otherks-T1pks	CP000473_c3: Solibacter usitatus Ellin6076, complete genome. (6% of genes show similarity).
	contig13939length=1097numreads=9	Arylpolyene	CP006877_c1: Rhizobium gallicum bv. gallicum R602, complete genome. (4% of genes show similarity).
ATII 1500	contig00004length=44710numreads=744	cf_putative	CP000249_c14: Frankia sp. CcI3, complete genome. (6% of genes show similarity).
	contig00007length=34963numreads=593	cf_putative	CP009747_c6: Pseudomonas parafulva strain CRS01-1, complete genome. (6% of genes show similarity).
	contig00009length=30341numreads=471	cf_putative	
	contig00041length=15646numreads=239	cf_putative	CXWD01000030_c1: Labrenzia alexandrii genome assembly L.alexandriiCECT5112 ... (14% of genes show similarity).
	contig00045length=15310numreads=230	cf_putative	
	contig00069length=12538numreads=153	cf_putative	AKJD01000052_c1: Pseudomonas sp. GM80 PMI37 contig70.70, whole genome shotg... (7% of genes show similarity).
	contig00084length=11158numreads=166	cf_putative	JWLJ01000001_c3: Ruegeria sp. ANG-R contig 1, whole genome shotgun sequence. (4% of genes show similarity).
	contig00094length=10415numreads=121	cf_putative	CP011497_c26: Streptomyces incarnatus strain NRRL 8089 sequence. (8% of genes show similarity).
	contig00101length=9926numreads=141	cf_putative	JJOB01000001_c18: Streptomyces sp. NTK 937 contig00001, whole genome shotgu... (4% of genes show similarity).

	contig00169length=6909numreads=102	cf_putative	AHFF01000022_c1: Bacillus cereus VD148 cont1.22, whole genome shotgun seque... (5% of genes show similarity).
	contig00186length=6639numreads=112	cf_putative	DS995276_c3: Rhodobacteraceae bacterium HTCC2083 scf 1106200183017 genomic ... (7% of genes show similarity).
	contig00200length=6334numreads=96	cf_putative	LJTN01000025_c1: Myxococcales bacterium SG8 38 WOR 8-12 590, whole genome s... (6% of genes show similarity).
	contig00515length=3819numreads=47	cf_putative	CP009438_c3: Streptomyces glaucescens strain GLA.O, complete genome. (5% of genes show similarity), **Homologous subcluster: AJ862840_2_c2: streptomycin dTDP-L-dihydrostreptose (40% of genes show similarity) and Homologous known gene cluster: BGC0000814: K-252a biosynthetic gene cluster (5% of genes show similarity).**
	contig00539length=3771numreads=71	cf_putative	No significant ClusterBlast hits found.
	contig01222length=2578numreads=15	cf_putative	No significant ClusterBlast hits found.
	contig06669length=1313numreads=4	Terpene	No significant ClusterBlast hits found.
	contig01294length=2533numreads=31	T3pks	No significant ClusterBlast hits found.
	contig00106length=9747numreads=151	Other	HE971709_c14: Streptomyces davawensis strain JCM 4913 complete genome. (3% of genes show similarity).
	contig00019length=23705numreads=385	Otherks-Pufa-T1pks	CP011456_c1: Anabaena sp. wa102 strain WA102, complete genome. (10% of genes show similarity) **and Homologous known gene cluster: BGC0000869: Heterocyst glycolipids biosynthetic gene cluster (28% of genes show similarity).**
	contig02435length=1971numreads=6	T2pks-Cf_fatty_acid	CP002552_c1: Nitrosomonas sp. AL212, complete genome. (8% of genes show similarity) **and Homologous known gene**

			**cluster: BGC0001382: Svaricin biosynthetic gene cluster (6% of genes show similarity).**
	contig02788length=1874numreads=7	Lantipeptide	
	contig00008length=15810numreads=415	cf_putative	
	contig00019length=12216numreads=219	cf_putative	LELK01000001_c4: Anaerobacillus macyae strain DSM 16346 super1, whole genom... (11% of genes show similarity).
	contig00045length=8834numreads=183	cf_putative	HE681424_c1: Taylorella asinigenitalis 14/45 draft genome. (12% of genes show similarity) **and Homologous known gene cluster: BGC0000760: Emulsan biosynthetic gene cluster (18% of genes show similarity).**
	contig01182length=1942numreads=17	Bacteriocin	LMQJ01000013_c1: Bradyrhizobium sp. Leaf396 contig 20, whole genome shotgun... (4% of genes show similarity).
	contig00034length=9414numreads=245	T3pks	CAHP01000020_c1: Phaeospirillum molischianum DSM 120 WGS project CAHP000000... (6% of genes show similarity).
	contig00699length=2506numreads=159	T3pks	No significant ClusterBlast hits found.
	contig00007length=16388numreads=481	Other	AYWO01000012_c1: Mesorhizobium sp. LNHC252B00 scaffold0012, whole genome sh... (7% of genes show similarity).
**ATII INF**	contig02792length=1234numreads=24	Other	No significant ClusterBlast hits found.
	contig00003length=39128numreads=2364	cf_putative	ADMS01000052_c1: Achromobacter piechaudii ATCC 43553 contig00055, whole gen... (10% of genes show similarity).
**ATII UCL**	contig00070length=13014numreads=187	cf_putative	

	contig00098length=10424numreads=209	cf_putative	AYWU01000002_c1: Mesorhizobium sp. L48C026A00 scaffold0002, whole genome sh... (20% of genes show similarity) **and Homologous known gene cluster: BGC0000774: Lipopolysaccharide biosynthetic gene cluster (5% of genes show similarity).**
	contig00181length=7318numreads=408	cf_putative	JFYX01000021_c1: Pseudomonas sp. RIT357 contigs28, whole genome shotgun seq... (14% of genes show similarity).
	contig00226length=6500numreads=82	cf_putative	CP007699_c33: Streptomyces lydicus A02, complete genome. (9% of genes show similarity), **Homologous subcluster: AJ006985_1_c1: 5'-hydroxystreptomycin CDP-N-methyl-L-glucosamine (40% of genes show similarity) and Homologous known gene cluster: BGC0000690: 5'-hydroxystreptomycin biosynthetic gene cluster (9% of genes show similarity).**
	contig00305length=5370numreads=252	cf_putative	
	contig01335length=2365numreads=32	Bacteriocin	CP005587_c3: Hyphomicrobium denitrificans 1NES1, complete genome. (8% of genes show similarity).
	contig00001length=103389numreads=5791	T3pks	JPKG01000033_c1: Bosea sp. LC85 contig39, whole genome shotgun sequence. (9% of genes show similarity).
	contig01020length=2708numreads=45	T3pks	No significant ClusterBlast hits found.
	contig00005length=36026numreads=1722	Other	CP003041_c6: Pseudomonas fluorescens A506, complete genome. (17% of genes show similarity) **and Homologous known gene cluster: BGC0000268: Sch47554 / Sch47555 biosynthetic gene cluster (3% of genes show similarity).**
	contig00300length=5408numreads=93	NRPS	CP005956_c1: Rhizobium etli bv. mimosae str. Mim1 plasmid pRetMIM1f, comple... (3% of genes show similarity) **and Homologous known gene cluster: BGC0000457: Vicibactin biosynthetic gene cluster (22% of genes show similarity).**
**ATII LCL**	contig00001length=350936numreads=72880	cf_putative	CP003389_c10: Corallococcus coralloides DSM 2259, complete genome. (5% of genes show similarity).

	contig00001length=350936numreads=72880 (another)	cf_putative	LGIS01000033_c2: Pantoea sp. RIT-PI-b NODE 2 length 623809 cov 27.3686 ID 3... (9% of genes show similarity).
	contig00001length=350936numreads=72880 (also another)	cf_putative	CCMY01000067_c1: Mesorhizobium sp. ORS3324 genome assembly Illumina assembl... (5% of genes show similarity).
	contig00003length=270539numreads=21524	cf_putative	LIUV01000026_c1: Pseudomonas sp. 655 contig 26, whole genome shotgun sequen... (12% of genes show similarity).
	contig00005length=217427numreads=22485	cf_putative	AYWU01000002_c1: Mesorhizobium sp. L48C026A00 scaffold0002, whole genome sh... (20% of genes show similarity) and **Homologous known gene cluster: BGC0000774: Lipopolysaccharide biosynthetic gene cluster (5% of genes show similarity).**
	contig00005length=217427numreads=22485 (another)	cf_putative	JMQM01000002_c1: Nitratireductor basaltis strain UMTGB225 contig000002, who... (10% of genes show similarity).
	contig00007length=206774numreads=15005	cf_putative	
	contig00008length=205649numreads=15032	cf_putative	BARX01000011_c1: Agarivorans albus MKT 106 DNA, contig: contig00011, whole ... (14% of genes show similarity).
	contig00008length=205649numreads=15032 (another)	cf_putative	AIMB01000003_c1: Bartonella tamiae Th239 cont1.3, whole genome shotgun sequ... (6% of genes show similarity).
	contig00009length=204526numreads=15731	cf_putative	DF820457_c2: Bacterium UASB14 DNA, scaffold: UASB14 scaffold 3, whole genom... (7% of genes show similarity).
	contig00009length=204526numreads=15732 (another)	cf_putative	DS995298_c1: Candidatus Pelagibacter sp. HTCC7211 scf 1105874033148 genomic... (4% of genes show similarity).

	contig00010length=204490numreads=18991	cf_putative	AYWX01000011_c1: Mesorhizobium sp. L2C084A000 scaffold0011, whole genome sh... (18% of genes show similarity).
	contig00010length=204490numreads=18991 (another)	cf_putative	BAND01000075_c1: Acidomonas methanolica NBRC 104435 DNA, contig: Amme 075, ... (12% of genes show similarity).
	contig00013length=177188numreads=11882	cf_putative	CP006880_c3: Rhizobium gallicum bv. gallicum R602 plasmid pRgalR602c, compl... (11% of genes show similarity).
	contig00013length=177188numreads=11882 (another)	cf_putative	AIMG01000004_c1: Bartonella tamiae Th307 cont1.4, whole genome shotgun sequ... (7% of genes show similarity).
	contig00013length=177188numreads=11882 (also another)	cf_putative	AKKA01000087_c1: Rhizobium sp. CF122 PMI09 contig154.154, whole genome shot... (4% of genes show similarity).
	contig00015length=143284numreads=26194	cf_putative	CP011501_c5: Pandoraea apista strain AU2161, complete genome. (6% of genes show similarity).
	contig00016length=142654numreads=7755	cf_putative	CP000633_c2: Agrobacterium vitis S4 chromosome 1, complete sequence. (10% of genes show similarity).
	contig00025length=100848numreads=20724	cf_putative	LMNZ01000020_c1: Rhizobium sp. Leaf311 contig 4, whole genome shotgun seque... (11% of genes show similarity).

	contig00035length=76695numreads=7821	cf_putative	CP003987_c23: Streptomyces sp. 769, complete genome. (6% of genes show similarity) **and Homologous known gene cluster: BGC0000093: Meilingmycin biosynthetic gene cluster (2% of genes show similarity).**
	contig00035length=76695numreads=7821 (also another)	cf_putative	LMEC01000001_c2: Massilia sp. Root418 contig 1, whole genome shotgun sequen... (6% of genes show similarity).
	contig00035length=76695numreads=7821 (another)	cf_putative	CP005094_c5: Azotobacter vinelandii CA, complete genome. (5% of genes show similarity).
	contig00037length=74628numreads=2831	cf_putative	JTDI01000008_c1: Novosphingobium malaysiense strain MUSC 273 Contig8, whole... (9% of genes show similarity).
	contig00048length=60613numreads=4313	cf_putative	CP000884_c2: Delftia acidovorans SPH-1, complete genome. (5% of genes show similarity).
	contig00052length=56204numreads=2513	cf_putative	
	contig00071length=42876numreads=2089	cf_putative	CP000075_c1: Pseudomonas syringae pv. syringae B728a, complete genome. (5% of genes show similarity).
	contig00082length=39278numreads=1132	cf_putative	No significant ClusterBlast hits found.
	contig00083length=38248numreads=1697	cf_putative	JXCB01000004_c6: Tolypothrix campylonemoides VB511288 scaffold 0, whole gen... (9% of genes show similarity).
	contig00109length=29318numreads=696	cf_putative	
	contig00125length=26339numreads=551	cf_putative	CP002878_c1: Cupriavidus necator N-1 chromosome 2, complete sequence. (15% of genes show similarity) **and Homologous known gene cluster: BGC0000015: Amphotericin biosynthetic gene cluster (23% of genes show similarity).**

	contig00134length=24742numreads=630	cf_putative	ALPV02000014_c1: Pseudomonas putida LS46 Contig14, whole genome shotgun seq... (16% of genes show similarity), **Homologous subclusters EU255259_2_c2: spectinomycin dTDP-actinospectose (66% of genes show similarity) and Homologous known gene cluster: BGC0000782: O-antigen biosynthetic gene cluster (38% of genes show similarity).**
	contig00139length=24270numreads=1936	cf_putative	CP006668_c2: Ralstonia pickettii DTP0602 chromosome 2, complete sequence. (11% of genes show similarity).
	contig00154length=22386numreads=345	cf_putative	HG916765_c3: Castellaniella defragrans 65Phen complete genome. (14% of genes show similarity).
	contig00159length=22125numreads=689	cf_putative	ALJE01000007_c1: Achromobacter piechaudii HLE Contig 7, whole genome shotgu... (7% of genes show similarity).
	contig00161length=21888numreads=606	cf_putative	JSWJ01000005_c1: Phaeobacter sp. S60 plasmid unnamed 1 Phaeobacter S60 cont... (6% of genes show similarity).
	contig00177length=19862numreads=349	cf_putative	AYLZ02000057_c1: Shinella sp. DD12 SHLA 70c, whole genome shotgun sequence. (29% of genes show similarity).
	contig00184length=19274numreads=279	cf_putative	FP885907_c3: Ralstonia solanacearum CFBP2957 plasmid RCFBPv3 mp, complete g... (6% of genes show similarity).
	contig00191length=18732numreads=843	cf_putative	AP012547_c1: Sulfuritalea hydrogenivorans sk43H DNA, complete genome. (10% of genes show similarity).
	contig00193length=18261numreads=7592	cf_putative	ADMS01000052_c1: Achromobacter piechaudii ATCC 43553 contig00055, whole gen... (13% of genes show similarity).
	contig00230length=16257numreads=739	cf_putative	CP000613_c4: Rhodospirillum centenum SW, complete genome. (11% of genes show similarity).
	contig00274length=14008numreads=282	cf_putative	AP014637_c1: Pseudomonas sp. StFLB209 DNA, complete genome. (9% of genes show similarity) **and Homologous known gene cluster: BGC0000925: Quinolobactin biosynthetic gene cluster (13% of genes show similarity).**

	as: contig00281length=13803numreads=324	cf_putative	AP012547_c1: Sulfuritalea hydrogenivorans sk43H DNA, complete genome. (4% of genes show similarity).
	contig00316length=11637numreads=205	cf_putative	CP001053_c7: Burkholderia phytofirmans PsJN chromosome 2, complete sequence. (5% of genes show similarity).
	contig00325length=11346numreads=6037	cf_putative	JFYX01000021_c1: Pseudomonas sp. RIT357 contigs28, whole genome shotgun seq... (14% of genes show similarity).
	contig00389length=9748numreads=235	cf_putative	No significant ClusterBlast hits found.
	contig00392length=9681numreads=106	cf_putative	LFDT01000002_c1: Vogesella sp. EB contig00002, whole genome shotgun sequence. (12% of genes show similarity).
	contig00786length=5579numreads=117	cf_putative	AKJC01000167_c1: Pseudomonas sp. GM84 PMI38 contig211.211, whole genome sho... (20% of genes show similarity).
	contig01684length=3333numreads=804	cf_putative	No significant ClusterBlast hits found.
	contig00006length=207406numreads=20770	Terpene	CP008820_c1: Ochrobactrum anthropi strain OAB chromosome 1, complete sequen... (43% of genes show similarity).
	contig00346length=10703numreads=181	Terpene	JFJV02000240_c1: Cupriavidus sp. SK-3 Scaffold240, whole genome shotgun seq... (38% of genes show similarity).
	contig00464length=8551numreads=103	Terpene	CP010537_c2: Cupriavidus basilensis strain 4G11 chromosome secondary, compl... (12% of genes show similarity).
	contig05112length=1606numreads=7	Terpene	ALIX01000357_c1: Burkholderia multivorans CF2 ctg100011134642, whole genome... (6% of genes show similarity).
	contig05848length=1463numreads=10	Terpene	LFZM01000006_c1: Ralstonia sp. MD27 contig 6, whole genome shotgun sequence. (5% of genes show similarity).

	contig00006length=207406numreads=20770	Bacteriocin	ADVZ01000001_c1: Afipia sp. 1NLS2 ctg00009, whole genome shotgun sequence. (21% of genes show similarity).
	contig00677length=6287numreads=77	Bacteriocin	AP014577_c3: Burkholderia sp. RPE67 DNA, complete genome, chromosome: 2. (13% of genes show similarity).
	contig01116length=4368numreads=60	Bacteriocin	LFZM01000037_c1: Ralstonia sp. MD27 contig 37, whole genome shotgun sequence. (7% of genes show similarity).
	contig00053length=54654numreads=25022	T3pks	LMKV01000001_c1: Sphingobium sp. Leaf26 contig 1, whole genome shotgun sequ... (11% of genes show similarity).
	contig01385length=3789numreads=87	T3pks	No significant ClusterBlast hits found.
	contig07333length=1261numreads=48	T3pks	No significant ClusterBlast hits found.
	contig00001length=350936numreads=72880	Other	JPRX01000001_c1: Pseudomonas sp. BRG-100 scaffold00001, whole genome shotgu... (24% of genes show similarity) and **Homologous known gene cluster: BGC0000268: Sch47554 / Sch47555 biosynthetic gene cluster (3% of genes show similarity).**
	contig00007length=206774numreads=15005	Cf_saccharide-nrps	JH719381_c5: Rhizobium leguminosarum bv. trifolii WSM597 genomic scaffold R... (37% of genes show similarity) and **Homologous known gene clusters: BGC0000457: Vicibactin biosynthetic gene cluster (77% of genes show similarity).**
	contig01728length=3279numreads=37		CP010537_c1: Cupriavidus basilensis strain 4G11 chromosome secondary, compl... (6% of genes show similarity) and **Homologous known gene cluster: BGC0000406: Phosphinothricin biosynthetic gene cluster (6% of genes show similarity).**
	contig00164length=21347numreads=606	T3pks-cf_saccharide	No significant ClusterBlast hits found.

	contig04718length=1699numreads=9	NRPS	AFJC01000014_c1: Microcoleus vaginatus FGP-2 ctg195, whole genome shotgun s... (17% of genes show similarity) and **Homologous known gene cluster: BGC0001189: Taiwachelin biosynthetic gene cluster (11% of genes show similarity).**
	contig04769length=1691numreads=23	NRPS	No significant ClusterBlast hits found.
	contig07600length=1231numreads=6	Arylpolyene	CP000353_c3: Cupriavidus metallidurans CH34 megaplasmid, complete sequence. (9% of genes show similarity).
	contig00025length=100848numreads=20724	Cf_fatty_acid-Cf_saccharide	
**DD BR**	contig01143length=1615numreads=20	Arylpolyene	CP006877_c1: Rhizobium gallicum bv. gallicum R602, complete genome. (4% of genes show similarity).
**DD INF**			
	contig00007length=10201numreads=689	Cf_putative	CP001348_c4: Clostridium cellulolyticum H10, complete genome. (5% of genes show similarity).
	contig00127length=4316numreads=85	Cf_putative	No significant ClusterBlast hits found.
	contig01013length=2026numreads=51	Bacteriocin	No significant ClusterBlast hits found.
	contig01029length=2018numreads=88	Bacteriocin	No significant ClusterBlast hits found.
**KD BR**	contig04134length=1186numreads=32	Bacteriocin	No significant ClusterBlast hits found.
**KD UINF**	contig00012length=32850numreads=1256	cf_putative	

	contig00041length=22104numreads=3404	cf_putative	CP002049_c1: Truepera radiovictrix DSM 17093, complete genome. (11% of genes show similarity).
	contig00049length=20767numreads=828	cf_putative	
	contig00060length=18728numreads=3399	cf_putative	CP007574_c23: Streptomyces albulus strain NK660, complete genome. (2% of genes show similarity).
	contig00066length=18433numreads=702	cf_putative	FR845719_c20: Streptomyces venezuelae ATCC 10712 complete genome. (4% of genes show similarity).
	contig00070length=17741numreads=688	cf_putative	FQ311869_c1: Azospirillum lipoferum 4B plasmid AZO p1 complete genome. (12% of genes show similarity).
	contig00083length=16750numreads=737	cf_putative	CP010976_c10: Paenibacillus sp. IHBB 10380, complete genome. (11% of genes show similarity).
	contig00089length=16132numreads=625	cf_putative	CP000724_c6: Alkaliphilus metalliredigens QYMF, complete genome. (5% of genes show similarity).
	contig00107length=14584numreads=654	cf_putative	CJWH01000005_c1: Salmonella enterica subsp. enterica serovar Typhi genome a... (4% of genes show similarity).
	contig00170length=12523numreads=513	cf_putative	CP001101_c5: Chlorobium phaeobacteroides BS1, complete genome. (4% of genes show similarity).
	contig00186length=11962numreads=1989	cf_putative	No significant ClusterBlast hits found.
	contig00210length=11215numreads=1580	cf_putative	CHTU01000002_c4: Streptococcus pneumoniae genome assembly 6755 6#9, scaffol... (7% of genes show similarity).
	contig00294length=9459numreads=405	cf_putative	AWSQ01000009_c1: Pseudomonas taeanensis MS-3 contig 09, whole genome shotgu... (6% of genes show similarity).

	contig00405length=8065numreads=150	cf_putative	No significant ClusterBlast hits found.
	contig00587length=6359numreads=78	cf_putative	CP000961_c2: Shewanella woodyi ATCC 51908, complete genome. (5% of genes show similarity).
	contig00717length=5461numreads=68	cf_putative	No significant ClusterBlast hits found.
	contig02003length=2786numreads=27	cf_putative	CXWC01000011_c1: Labrenzia alba genome assembly L.albaCECT5096 Spades Prokk... (4% of genes show similarity) and **Homologous known gene cluster: BGC0000780: O&K-antigen biosynthetic gene cluster (3% of genes show similarity).**
	contig02976length=2179numreads=34	Terpene	No significant ClusterBlast hits found.
	contig04985length=1630numreads=13	Terpene	LIGC01000002_c1: Mariprofundus ferrooxydans strain JV-1 Contig 2, whole gen... (4% of genes show similarity).
	contig11899length=1028numreads=24	Terpene	No significant ClusterBlast hits found.
	contig02994length=2172numreads=49	Bacteriocin	No significant ClusterBlast hits found.
	contig11953length=1020numreads=31	Bacteriocin	No significant ClusterBlast hits found.
	contig02012length=2784numreads=59	Other	No significant ClusterBlast hits found.
	contig00103length=14761numreads=323	NRPS	HE617159_c8: Bacillus amyloliquefaciens subsp. plantarum CAU B946 complete ... (10% of genes show similarity).

	contig01513length=3311numreads=80	NRPS	LIRN01000031_c1: Hapalosiphon sp. MRB220 contig 14, whole genome shotgun se... (10% of genes show similarity).
	contig04841length=1663numreads=49	NRPS	CAIH01000020_c1: Microcystis aeruginosa 9432 WGS project CAIH01000000 data,... (38% of genes show similarity).
	contig06330length=1445numreads=10	Microcin	
	contig04095length=1820numreads=16	Lantipeptide	
	contig00058length=19234numreads=452	Nrps-T1pks	CP012109_c13: Myxococcus sp. (contaminant ex DSM 436), complete genome. (9% of genes show similarity) and **Homologous known gene cluster: BGC0001125: Puwainaphycins biosynthetic gene cluster (50% of genes show similarity).**
	contig00660length=5849numreads=146	T1PKS	No significant ClusterBlast hits found.
	contig00850length=4867numreads=88	Ladderane-Cf_fatty_acid	AF324838_c1: Streptomyces antibioticus simocyclinone biosynthetic gene clus... (6% of genes show similarity).
	contig01825length=2949numreads=71	Ladderane-Cf_fatty_acid	ASST01000028_c1: Lachnospiraceae bacterium 3-1 acPFp-supercont1.11.C28, who... (7% of genes show similarity).
	contig01495length=3334numreads=147	Ectoine	AAOW01000022_c1: Neptuniibacter caesariensis strain MED92 1099521380312, wh... (9% of genes show similarity) and **Homologous known gene cluster: BGC0000853: Ectoine biosynthetic gene cluster (75% of genes show similarity).**
	contig04056length=1823numreads=28	Ectoine	JZKJ01000058_c1: Nitrospina sp. SCGC AAA799 C22 AAA799C22 61, whole genome ... (30% of genes show similarity) and **Homologous known gene cluster: BGC0000853: Ectoine biosynthetic gene cluster (50% of genes show similarity).**

	contig00227length=6473numreads=148	Cf_putative	AYSC01000002_c1: Blastomonas sp. CACIA14H2 contig00007, whole genome shotgu... (4% of genes show similarity).
	contig00288length=5980numreads=62	Cf_putative	JWSY01000020_c1: Brevundimonas nasdae strain TPW30 Contig 20, whole genome ... (4% of genes show similarity).
	contig00388length=5342numreads=102	Cf_putative	AZLW01000062_c1: Intestinibacter bartlettii DORA 8 9 Q606 CBAC00062, whole ... (16% of genes show similarity).
	contig00397length=5292numreads=102	Cf_putative	No significant ClusterBlast hits found.
	contig00881length=3779numreads=33	Cf_putative	AEJC01000250_c1: Streptomyces ipomoeae 91-03 gcontig 1108499723643, whole g... (10% of genes show similarity).
	contig02212length=2483numreads=43	Cf_putative	JDST02000018_c1: Candidatus Accumulibacter sp. SK-02 contig000023, whole ge... (5% of genes show similarity) and **Homologous known gene cluster: BGC0001411: Polysaccharide B biosynthetic gene cluster (6% of genes show similarity).**
	contig00631length=4362numreads=108	Bacteriocin	No significant ClusterBlast hits found.
	contig00714length=4147numreads=255	Bacteriocin	No significant ClusterBlast hits found.
	contig02043length=2578numreads=31	Bacteriocin	LCCT01000013_c1: Parcubacteria bacterium GW2011 GWE2 42 14 UU99 C0013, whol... (28% of genes show similarity).
	contig03387length=2064numreads=29	Bacteriocin	No significant ClusterBlast hits found.
**KD LINF**	contig04566length=1794numreads=38	Bacteriocin	No significant ClusterBlast hits found.

	contig06173length=1562numreads=99	Bacteriocin	No significant ClusterBlast hits found.
	contig03938length=1929numreads=30	Other	No significant ClusterBlast hits found.
	contig05383length=1666numreads=29	Other	No significant ClusterBlast hits found.
	contig00256length=6236numreads=111	NRPS	AAXW01000011_c1: Cyanothece sp. CCY0110 1101676644580, whole genome shotgun... (3% of genes show similarity).
	contig00627length=4381numreads=83	NRPS	JYPH01000039_c1: Bacillus cereus strain F528-94 contig278, whole genome sho... (7% of genes show similarity).
	contig03753length=1973numreads=62	NRPS	AHCL02000037_c1: Bacillus cereus VD142 actaa-supercont2.6.C37, whole genome... (7% of genes show similarity).
	contig06562length=1522numreads=41	NRPS	No significant ClusterBlast hits found.
	contig00161length=7333numreads=224	Ectoine	AAOW01000022_c1: Neptuniibacter caesariensis strain MED92 1099521380312, wh... (9% of genes show similarity) and **Homologous known gene cluster: BGC0000853: Ectoine biosynthetic gene cluster (75% of genes show similarity).**
	contig00200length=6749numreads=188	Cf_saccharide-Bacteriocin	No significant ClusterBlast hits found.
	contig00349length=5617numreads=624	Cf_saccharide-Bacteriocin	No significant ClusterBlast hits found.
	contig00587length=4536numreads=58	Cf_saccharide-Bacteriocin	No significant ClusterBlast hits found.
	contig05890length=1589numreads=7	Hserlactone	No significant ClusterBlast hits found.

	contig02165length=2229numreads=23	Cf_putative	CP001124_c5: Geobacter bemidjiensis Bem, complete genome. (5% of genes show similarity).
	contig02247length=2198numreads=40	Cf_putative	No significant ClusterBlast hits found.
	contig00013length=12599numreads=159	Cf_putative	No significant ClusterBlast hits found.
	contig00021length=10365numreads=126	Cf_putative	No significant ClusterBlast hits found.
	contig00030length=9342numreads=145	Cf_putative	CP000951_c1: Synechococcus sp. PCC 7002, complete genome. (5% of genes show similarity).
	contig00336length=4140numreads=47	Cf_putative	CP011941_c1: Synechococcus sp. WH 8020 genome. (4% of genes show similarity).
	contig00427length=3856numreads=79	Cf_putative	No significant ClusterBlast hits found.
	contig00505length=3681numreads=49	Cf_putative	No significant ClusterBlast hits found.
	contig00811length=3142numreads=80	Cf_putative	No significant ClusterBlast hits found.
	contig01090length=2796numreads=34	Cf_putative	LMVE01000134_c1: Cylindrospermopsis sp. CR12 cyanobacteria contig 90, whole... (9% of genes show similarity).
	contig07477length=1393numreads=13	Terpene	JNAS01000002_c3: Prochlorococcus marinus str. SB Contig35, whole genome sho... (7% of genes show similarity).
**ATII SDM**	contig00144length=5654numreads=69	Terpene	JNAO01000009_c1: Prochlorococcus marinus str. MIT 9314 contig00011, whole g... (13% of genes show similarity).

	contig12934length=1067numreads=11	Terpene	CP000576_c1: Prochlorococcus marinus str. MIT 9301, complete genome. (4% of genes show similarity).
	contig03820length=1779numreads=12	Terpene	CAKW01000091_c1: Cronobacter condimenti 1330 WGS project CAKW00000000 data,... (9% of genes show similarity).
	contig04553length=1666numreads=13	Bacteriocin	HF680312_c1: Thalassolituus oleivorans MIL-1 complete genome. (7% of genes show similarity).
	contig02814length=2028numreads=14	T3pks	CP011125_c6: Sandaracinus amylolyticus strain DSM 53668, complete genome. (4% of genes show similarity).
	contig00785length=3121numreads=39	Arylpolyene - cf_fatty_acid	CP006877_c1: Rhizobium gallicum bv. gallicum R602, complete genome. (4% of genes show similarity).
**DD SDM**	contig00023length=14210numreads=245	Cf_putative	No significant ClusterBlast hits found.
	contig00118length=7210numreads=102	Cf_putative	No significant ClusterBlast hits found.
	contig00149length=6666numreads=91	Cf_putative	CNAJ01000002_c1: Streptococcus pneumoniae genome assembly 6930 7#10, scaffo... (5% of genes show similarity).
	contig00255length=5445numreads=111	Cf_putative	No significant ClusterBlast hits found.
	contig00348length=4904numreads=168	Cf_putative	ALQA01000033_c1: Mycobacterium vaccae ATCC 25954 Contig033, whole genome sh... (5% of genes show similarity).
	contig00444length=4434numreads=69	Cf_putative	CP002638_c16: Verrucosispora maris AB-18-032, complete genome. (2% of genes show similarity).
	contig00462length=4379numreads=53	Cf_putative	LGEE01000248_c1: Streptomyces sp. NRRL F-6491 P443contig64.1, whole genome ... (7% of genes show similarity).

	contig00490length=4304numreads=164	Cf_putative	No significant ClusterBlast hits found.
	contig00544length=4129numreads=81	Cf_putative	CP003945_c1: Halothece sp. PCC 7418, complete genome. (4% of genes show similarity).
	contig00934length=3438numreads=63	Cf_putative	CP000825_c5: Prochlorococcus marinus str. MIT 9215, complete genome. (4% of genes show similarity).
	contig01296length=3087numreads=62	Cf_putative	No significant ClusterBlast hits found.
	contig01688length=2785numreads=46	Cf_putative	LMVE01000134_c1: Cylindrospermopsis sp. CR12 cyanobacteria contig 90, whole... (9% of genes show similarity).
	contig01837length=2720numreads=25	Cf_putative	No significant ClusterBlast hits found.
	contig01902length=2668numreads=30	Cf_putative	No significant ClusterBlast hits found.
	contig00999length=3370numreads=73	Terpene	CP000576_c3: Prochlorococcus marinus str. MIT 9301, complete genome. (10% of genes show similarity).
	contig10118length=1388numreads=7	Terpene	No significant ClusterBlast hits found.
	contig15912length=1110numreads=2	Terpene	CAQJ01000004_c1: Nitrospina gracilis 3/211 WGS project CAQJ00000000 data, c... (7% of genes show similarity).
	contig18228length=1026numreads=7	Terpene	No significant ClusterBlast hits found.
**NB SDM**	contig00117length=3583numreads=51	Cf_putative	No significant ClusterBlast hits found.

	contig00190length=3103numreads=23	Cf_putative	CP003548_c4: Nostoc sp. PCC 7107, complete genome. (4% of genes show similarity).
	contig00237length=2926numreads=21	Cf_putative	CP003946_c6: Leptolyngbya sp. PCC 7376, complete genome. (4% of genes show similarity).
	contig01215length=1638numreads=12	Terpene	JNAL01000014_c1: Prochlorococcus marinus str. MIT 9201 contig00008, whole g... (4% of genes show similarity).
	contig02059length=1319numreads=11	Terpene	

Table S9: Table showing the percentage of each SMGC as compared to the total SMGCs detected per site among the Red Sea brine samples.

Sites	ATII 50	ATII 200	ATII 700	ATII 1500	ATII SDM	DD SDM	NB SDM	DD BR	DD INF	ATII INF	ATII UCL	ATII LCL	KD BR	KD LINF	KD UINF
**Arylpolyene**	3.42	0.00	0.37	0.00	0.00	0.00	0.00	1.37	0.00	0.00	0.00	0.19	0.00	0.00	0.00
**Arylpolyene-cf_fatty_acid**	0.68	0.00	0.37	0.00	1.43	0.00	0.00	0.00	0.00	0.00	0.00	0.00	0.00	0.00	0.00
**Bacteriocin**	0.00	0.00	0.00	0.00	1.43	0.00	0.00	0.00	0.00	0.62	0.48	0.57	2.01	1.80	0.79
**Cf_fatty_acid**	14.38	17.06	10.41	10.71	10.00	1.75	6.25	0.00	0.00	1.23	4.76	2.10	1.34	0.90	8.73
**Cf_fatty_acid-Arylpolyene**	0.00	0.59	0.00	0.00	0.00	0.00	0.00	0.00	0.00	0.00	0.00	0.00	0.00	0.00	0.00
**Cf_fatty_acid-Cf_saccharide**	0.00	0.00	0.00	0.00	0.00	0.00	0.00	0.00	0.00	0.00	0.00	0.19	0.00	0.00	0.00
**Cf_putative**	12.33	5.29	11.52	8.93	14.29	12.28	18.75	0.00	0.00	1.85	2.86	8.59	1.34	1.80	6.75
**Cf_saccharide**	58.22	74.71	76.21	76.79	65.71	82.46	62.50	98.63	100.00	93.83	90.00	85.69	95.30	92.51	77.78
**Cf_saccharide-Bacteriocin**	0.00	0.00	0.00	0.00	0.00	0.00	0.00	0.00	0.00	0.00	0.00	0.00	0.00	0.90	0.00
**Cf_saccharide-nrps**	0.00	0.00	0.00	0.00	0.00	0.00	0.00	0.00	0.00	0.00	0.00	0.19	0.00	0.00	0.00
**Ectoine**	0.00	0.00	0.00	0.00	0.00	0.00	0.00	0.00	0.00	0.00	0.00	0.00	0.00	0.30	0.79
**Hserlactone**	0.00	0.00	0.00	0.00	0.00	0.00	0.00	0.00	0.00	0.00	0.00	0.00	0.00	0.30	0.00
**Ladderane-Cf_fatty_acid**	0.00	0.00	0.00	0.00	0.00	0.00	0.00	0.00	0.00	0.00	0.00	0.00	0.00	0.00	0.79
**Lantipeptide**	0.00	0.00	0.00	0.60	0.00	0.00	0.00	0.00	0.00	0.00	0.00	0.00	0.00	0.00	0.40
**Microcin**	0.68	0.59	0.00	0.00	0.00	0.00	0.00	0.00	0.00	0.00	0.00	0.00	0.00	0.00	0.40
**NRPS**	0.00	0.00	0.00	0.00	0.00	0.00	0.00	0.00	0.00	0.00	0.48	0.38	0.00	0.90	1.19

**Nrps-T1pks**	0.00	0.00	0.00	0.00	0.00	0.00	0.00	0.00	0.00	0.00	0.00	0.00	0.00	0.00	0.40
**Other**	0.00	0.59	0.37	0.60	0.00	0.00	0.00	0.00	0.00	1.23	0.48	0.19	0.00	0.60	0.40
**OtherKS**	0.68	0.59	0.00	0.00	0.00	0.00	0.00	0.00	0.00	0.00	0.00	0.00	0.00	0.00	0.00
**Otherks-Pufa**	0.00	0.00	0.37	0.00	0.00	0.00	0.00	0.00	0.00	0.00	0.00	0.00	0.00	0.00	0.00
**Otherks-Pufa-T1pks**	0.00	0.00	0.00	0.60	0.00	0.00	0.00	0.00	0.00	0.00	0.00	0.00	0.00	0.00	0.00
**Otherks-T1pks**	0.00	0.00	0.37	0.00	0.00	0.00	0.00	0.00	0.00	0.00	0.00	0.00	0.00	0.00	0.00
**Phosphonate**	0.00	0.00	0.00	0.00	0.00	0.00	0.00	0.00	0.00	0.00	0.00	0.19	0.00	0.00	0.00
**T1PKS**	0.00	0.00	0.00	0.00	0.00	0.00	0.00	0.00	0.00	0.00	0.00	0.00	0.00	0.00	0.40
**T2pks-Cf_fatty_acid**	0.00	0.00	0.00	0.60	0.00	0.00	0.00	0.00	0.00	0.00	0.00	0.00	0.00	0.00	0.00
**T3pks**	0.00	0.00	0.00	0.60	1.43	0.00	0.00	0.00	0.00	1.23	0.95	0.57	0.00	0.00	0.00
**T3pks-cf_saccharide**	0.00	0.00	0.00	0.00	0.00	0.00	0.00	0.00	0.00	0.00	0.00	0.19	0.00	0.00	0.00
**Terpene**	9.59	0.59	0.00	0.60	5.71	3.51	12.50	0.00	0.00	0.00	0.00	0.95	0.00	0.00	1.19

Table S10: All the detected archaeal and bacterial genera in all sites included in the dataset.

Archaeal / bacterial genera common to both Red Sea & non-Red Sea dataset	Archaeal / bacterial genera present only in the Red Sea dataset	Archaeal / bacterial genera present only in the non-Red Sea dataset
Acaryochloris	Abiotrophia	unclassified (derived from Thiotrichales)
Acetivibrio	Acetohalobium	
Acetobacter	Acholeplasma	
Achromobacter	Acidilobus	
Acidaminococcus	Actinomyces	
Acidimicrobium	Alicyclobacillus	
Acidiphilium	Aminobacterium	
Acidithiobacillus	Ammonifex	

Acidobacterium	Anaerofustis
Acidothermus	Anaerostipes
Acidovorax	Anaerotruncus
Aciduliprofundum	Anaplasma
Acinetobacter	Arcanobacterium
Actinobacillus	Bifidobacterium
Actinosynnema	Blautia
Aerococcus	Brevundimonas
Aeromicrobium	Butyrivibrio
Aeromonas	Caldicellulosiruptor
Aeropyrum	Caldivirga
Afipia	Candidatus Azobacteroides
Aggregatibacter	Candidatus Blochmannia
Agrobacterium	Candidatus Carsonella
Ahrensia	Candidatus Desulforudis
Akkermansia	Candidatus Korarchaeum
Albidiferax	Candidatus Liberibacter
Alcanivorax	Candidatus Zinderia

Algoriphagus	Catenibacterium	
Alicycliphilus	Cellulomonas	
Aliivibrio	Cellulosilyticum	
Alistipes	Citromicrobium	
Alkalilimnicola	Conexibacter	
Alkaliphilus	Coprobacillus	
Allochromatium	Coprococcus	
Alteromonas	Cryptobacterium	
Aminomonas	Cylindrospermopsis	
Amycolatopsis	Dehalogenimonas	
Anabaena	Dermacoccus	
Anaerobaculum	Desulfurococcus	
Anaerococcus	Dethiosulfovibrio	
Anaeromyxobacter	Dialister	
Anoxybacillus	Dorea	
Aquifex	Ehrlichia	
Archaeoglobus	Elusimicrobium	
Arcobacter	Epulopiscium	
Aromatoleum	Eremococcus	
Arthrobacter	Erysipelothrix	
Arthrospira	Ethanoligenens	

Asticcacaulis	Exiguobacterium	
Atopobium	Faecalibacterium	
Aurantimonas	Ferroglobus	
Azoarcus	Ferroplasma	
Azorhizobium	Fervidobacterium	
Azospirillum	Filifactor	
Azotobacter	Gardnerella	
Bacillus	Gemella	
Bacteroides	Glaciecola	
Bartonella	Gordonia	
Basfia	Granulibacter	
Bdellovibrio	Granulicatella	
Beggiatoa	Halalkalicoccus	
Beijerinckia	Haloarcula	
Bermanella	Halobacterium	
Beutenbergia	Haloferax	
Blastopirellula	Halogeometricum	
Blattabacterium	Halomicrobium	
Bordetella	Haloquadratum	
Borrelia	Halorhabdus	
Brachybacterium	Halorubrum	

Brachyspira	Haloterrigena	
Bradyrhizobium	Heliobacterium	
Brevibacillus	Holdemania	
Brevibacterium	Hydrogenobacter	
Brucella	Hyperthermus	
Buchnera	Ignisphaera	
Burkholderia	Intrasporangium	
Caldanaerobacter	Ketogulonicigenium	
Calditerrivibrio	Kribbella	
Caminibacter	Kytococcus	
Campylobacter	Lactococcus	
Candidatus Accumulibacter	Leifsonia	
Candidatus Amoebophilus	Lysinibacillus	
Candidatus Cloacamonas	Macrococcus	
Candidatus Hamiltonella	Megasphaera	
Candidatus Koribacter	Methanocella	
Candidatus Pelagibacter	Methanoculleus	
Candidatus Phytoplasma	Methanohalobium	
Candidatus Protochlamydia	Methanohalophilus	

Candidatus Puniceispirillum	Methanoplanus
Candidatus Regiella	Methanosaeta
Candidatus Solibacter	Methanosphaerula
Candidatus Sulcia	Methanothermococcus
Capnocytophaga	Methanothermus
Carboxydothermus	Microcystis
Cardiobacterium	Mitsuokella
Catenulispora	Mobiluncus
Caulobacter	Natrialba
Cellvibrio	Neorickettsia
Cenarchaeum	Nocardiopsis
Chelativorans	Oceanithermus
Chitinophaga	Oenococcus
Chlamydia	Oribacterium
Chlamydophila	Orientia
Chlorobaculum	Parascardovia
Chlorobium	Parvularcula
Chloroflexus	Pediococcus
Chloroherpeton	Peptoniphilus
Chromobacterium	Petrotoga

Chromohalobacter	Phenylobacterium
Chryseobacterium	Propionibacterium
Chthoniobacter	Pyramidobacter
Citreicella	Renibacterium
Citrobacter	Rickettsiella
Clavibacter	Roseburia
Clostridium	Rothia
Collinsella	Sanguibacter
Colwellia	Segniliparus
Comamonas	Shuttleworthia
Congregibacter	Slackia
Coprothermobacter	Stackebrandtia
Coraliomargarita	Staphylothermus
Corynebacterium	Streptobacillus
Coxiella	Subdoligranulum
Croceibacter	Terriglobus
Crocosphaera	Thermocrinis
Cronobacter	Thermoplasma
Cupriavidus	Thermoproteus
Cyanobium	Thermosinus
Cyanothece	Thermosphaera

Cytophaga	Tsukamurella
Dechloromonas	Turicibacter
Deferribacter	unclassified (derived from Actinobacteria (class))
Dehalococcoides	unclassified (derived from Bacteria)
Deinococcus	unclassified (derived from Chroococcales)
Delftia	unclassified (derived from Clostridiales Family XI. Incertae Sedis)
Denitrovibrio	unclassified (derived from Clostridiales)
Desulfarculus	unclassified (derived from Erysipelotrichaceae)
Desulfatibacillum	unclassified (derived from Euryarchaeota)
Desulfitobacterium	unclassified (derived from Lachnospiraceae)
Desulfobacterium	unclassified (derived from Thermotogales)
Desulfococcus	Ureaplasma

Desulfohalobium	Vulcanisaeta
Desulfomicrobium	Weissella
Desulfonatronospira	Wigglesworthia
Desulfotalea	Xylanimonas
Desulfotomaculum	Xylella
Desulfovibrio	
Desulfurispirillum	
Desulfurivibrio	
Desulfuromonas	
Dethiobacter	
Dichelobacter	
Dickeya	
Dictyoglomus	
Dinoroseobacter	
Dokdonia	
Dyadobacter	
Edwardsiella	
Eggerthella	
Eikenella	
Endoriftia	
Enhydrobacter	

Enterobacter	
Enterococcus	
Erwinia	
Erythrobacter	
Escherichia	
Eubacterium	
Ferrimonas	
Fibrobacter	
Finegoldia	
Flavobacterium	
Francisella	
Frankia	
Fulvimarina	
Fusobacterium	
Gallionella	
Gemmata	
Gemmatimonas	
Geobacillus	
Geobacter	
Geodermatophilus	
Gloeobacter	

Gluconacetobacter
Gluconobacter
Gramella
Haemophilus
Hahella
Halanaerobium
Haliangium
Halomonas
Halorhodospira
Halothermothrix
Halothiobacillus
Helicobacter
Herbaspirillum
Herminiimonas
Herpetosiphon
Hirschia
Histophilus
Hoeflea
Hydrogenivirga
Hydrogenobaculum
Hyphomicrobium

Hyphomonas	
Idiomarina	
Ignicoccus	
Ilyobacter	
Janibacter	
Jannaschia	
Janthinobacterium	
Jonesia	
Jonquetella	
Kangiella	
Kineococcus	
Kingella	
Klebsiella	
Kocuria	
Kordia	
Kosmotoga	
Ktedonobacter	
Labrenzia	
Lactobacillus	
Laribacter	
Lawsonia	

Leadbetterella
Leeuwenhoekiella
Legionella
Lentisphaera
Leptospira
Leptothrix
Leptotrichia
Leuconostoc
Limnobacter
Listeria
Loktanella
Lutiella
Lyngbya
Magnetococcus
Magnetospirillum
Mannheimia
Maribacter
Maricaulis
Marinobacter
Marinomonas
Mariprofundus

Maritimibacter
Marivirga
Meiothermus
Mesorhizobium
Metallosphaera
Methanobrevibacter
Methanocaldococcus
Methanococcoides
Methanococcus
Methanocorpusculum
Methanopyrus
Methanoregula
Methanosarcina
Methanosphaera
Methanospirillum
Methanothermobacter
Methylacidiphilum
Methylibium
Methylobacillus
Methylobacter
Methylobacterium

Methylocella	
Methylococcus	
Methylophaga	
Methylosinus	
Methylotenera	
Methylovorus	
Micrococcus	
Microcoleus	
Micromonospora	
Microscilla	
Moorella	
Moraxella	
Moritella	
Mucilaginibacter	
Mycobacterium	
Mycoplasma	
Myxococcus	
Nakamurella	
Nanoarchaeum	
Natranaerobius	
Natronomonas	

Nautilia
Neisseria
Neptuniibacter
Nitratiruptor
Nitrobacter
Nitrococcus
Nitrosococcus
Nitrosomonas
Nitrosopumilus
Nitrosospira
Nitrospira
Nocardia
Nocardioides
Nodularia
Nostoc
Novosphingobium
Oceanibulbus
Oceanicaulis
Oceanicola
Oceanobacillus
Ochrobactrum

Octadecabacter
Oligotropha
Olsenella
Opitutus
Oscillatoria
Oscillochloris
Oxalobacter
Paenibacillus
Paludibacter
Pantoea
Parabacteroides
Parachlamydia
Paracoccus
Parvibaculum
Pasteurella
Pectobacterium
Pedobacter
Pelagibaca
Pelobacter
Pelodictyon
Pelotomaculum

Peptostreptococcus	
Persephonella	
Phaeobacter	
Photobacterium	
Photorhabdus	
Picrophilus	
Pirellula	
Planctomyces	
Plesiocystis	
Polaribacter	
Polaromonas	
Polynucleobacter	
Porphyromonas	
Prevotella	
Prochlorococcus	
Prosthecochloris	
Proteus	
Providencia	
Pseudoalteromonas	
Pseudomonas	
Pseudoramibacter	

Pseudovibrio	
Psychrobacter	
Psychroflexus	
Psychromonas	
Pyrobaculum	
Pyrococcus	
Ralstonia	
Raphidiopsis	
Reinekea	
Rhizobium	
Rhodobacter	
Rhodococcus	
Rhodomicrobium	
Rhodopirellula	
Rhodopseudomonas	
Rhodospirillum	
Rhodothermus	
Rickettsia	
Riemerella	
Robiginitalea	
Roseibium	

Roseiflexus
Roseobacter
Roseomonas
Roseovarius
Rubrobacter
Ruegeria
Ruminococcus
Saccharomonospora
Saccharophagus
Saccharopolyspora
Sagittula
Salinibacter
Salinispora
Salmonella
Sebaldella
Selenomonas
Serratia
Shewanella
Shigella
Sideroxydans
Simonsiella

Sinorhizobium	
Sodalis	
Sorangium	
Sphaerobacter	
Sphingobacterium	
Sphingobium	
Sphingomonas	
Sphingopyxis	
Spirochaeta	
Spirosoma	
Staphylococcus	
Starkeya	
Stenotrophomonas	
Stigmatella	
Streptococcus	
Streptomyces	
Streptosporangium	
Sulfitobacter	
Sulfolobus	
Sulfuricurvum	
Sulfurihydrogenibium	

Sulfurimonas	
Sulfurospirillum	
Sulfurovum	
Symbiobacterium	
Synechococcus	
Synechocystis	
Syntrophobacter	
Syntrophomonas	
Syntrophothermus	
Syntrophus	
Teredinibacter	
Thalassobium	
Thauera	
Thermaerobacter	
Thermanaerovibrio	
Thermincola	
Thermoanaerobacter	
Thermoanaerobacterium	
Thermobaculum	
Thermobifida	
Thermobispora	

Thermococcus		
Thermodesulfovibrio		
Thermofilum		
Thermomicrobium		
Thermomonospora		
Thermosediminibacter		
Thermosipho		
Thermosynechococcus		
Thermotoga		
Thermus		
Thioalkalivibrio		
Thiobacillus		
Thiomicrospira		
Thiomonas		
Tolumonas		
Treponema		
Trichodesmium		
Tropheryma		
Truepera		
unclassified (derived from Alicyclobacillaceae)		

unclassified (derived from Alphaproteobacteria)	
unclassified (derived from Alteromonadales)	
unclassified (derived from Bacteroidetes)	
unclassified (derived from Betaproteobacteria)	
unclassified (derived from Burkholderiales)	
unclassified (derived from Campylobacterales)	
unclassified (derived from Candidatus Poribacteria)	
unclassified (derived from Deltaproteobacteria)	
unclassified (derived from Elusimicrobia)	
unclassified (derived from Flavobacteria)	
unclassified (derived from Flavobacteriaceae)	
unclassified (derived from Flavobacteriales)	

unclassified (derived from Gammaproteobacteria)	
unclassified (derived from Methylophilales)	
unclassified (derived from Opitutaceae)	
unclassified (derived from Rhodobacteraceae)	
unclassified (derived from Rhodobacterales)	
unclassified (derived from Rickettsiales)	
unclassified (derived from Ruminococcaceae)	
unclassified (derived from Verrucomicrobia subdivision 3)	
unclassified (derived from Verrucomicrobiales)	
unclassified (derived from Vibrionaceae)	
unclassified (derived from Vibrionales)	
Variovorax	
Veillonella	

Verminephrobacter	
Verrucomicrobium	
Vibrio	
Victivallis	
Waddlia	
Wolbachia	
Wolinella	
Xanthobacter	
Xanthomonas	
Xenorhabdus	
Yersinia	
Zunongwangia	
Zymomonas	
